# Modulation of the NO-cGMP pathway has no effect on olfactory responses in the *Drosophila* antenna

**DOI:** 10.3389/fncel.2023.1180798

**Published:** 2023-05-25

**Authors:** Sinisa Prelic, Merid N. Getahun, Sabine Kaltofen, Bill S. Hansson, Dieter Wicher

**Affiliations:** ^1^Department of Evolutionary Neuroethology, Max Planck Institute for Chemical Ecology, Jena, Germany; ^2^International Centre of Insect Physiology and Ecology, Nairobi, Kenya

**Keywords:** nitric oxide, calcium imaging, single sensillum recording, insect olfaction, olfactory sensory neuron, cGMP, cAMP, cyclic nucleotide

## Abstract

Olfaction is a crucial sensory modality in insects and is underpinned by odor-sensitive sensory neurons expressing odorant receptors that function in the dendrites as odorant-gated ion channels. Along with expression, trafficking, and receptor complexing, the regulation of odorant receptor function is paramount to ensure the extraordinary sensory abilities of insects. However, the full extent of regulation of sensory neuron activity remains to be elucidated. For instance, our understanding of the intracellular effectors that mediate signaling pathways within antennal cells is incomplete within the context of olfaction *in vivo*. Here, with the use of optical and electrophysiological techniques in live antennal tissue, we investigate whether nitric oxide signaling occurs in the sensory periphery of *Drosophila*. To answer this, we first query antennal transcriptomic datasets to demonstrate the presence of nitric oxide signaling machinery in antennal tissue. Next, by applying various modulators of the NO-cGMP pathway in open antennal preparations, we show that olfactory responses are unaffected by a wide panel of NO-cGMP pathway inhibitors and activators over short and long timescales. We further examine the action of cAMP and cGMP, cyclic nucleotides previously linked to olfactory processes as intracellular potentiators of receptor functioning, and find that both long-term and short-term applications or microinjections of cGMP have no effect on olfactory responses *in vivo* as measured by calcium imaging and single sensillum recording. The absence of the effect of cGMP is shown in contrast to cAMP, which elicits increased responses when perfused shortly before olfactory responses in OSNs. Taken together, the apparent absence of nitric oxide signaling in olfactory neurons indicates that this gaseous messenger may play no role as a regulator of olfactory transduction in insects, though may play other physiological roles at the sensory periphery of the antenna.

## 1. Introduction

The sense of smell is a crucial sensory modality in insects as it underpins a variety of basic and complex foraging, sexual, social, and survival behaviors such as locating and assessing food sources, mating partners, and locations for oviposition and aggregation, as well as the avoiding predators, parasitoids, and biotic and abiotic dangers (Vosshall, 2000).

Neopteran insects possess a family of chemoreceptors termed odorant receptors (ORs), the broadest of three distinct types of chemosensory receptors (Gomez-Diaz et al., 2018), which serve to receive volatile chemical signals and transduce this sensory information into neuronal activity (Wicher, 2018; Wicher and Miazzi, 2021). ORs are primarily expressed in olfactory sensory neurons (OSNs) where they are trafficked into the dendritic segments that innervate hair-like structures called sensilla (Dobritsa et al., 2003; Larsson et al., 2004; Benton et al., 2006; Bahk and Jones, 2016). Olfactory sensilla are most abundant on the antenna, a major olfactory appendage in insects, and are distributed in a highly stereotyped and genetically determined fashion, such that one-or-few OSNs each expressing only one-or-few ORs innervate a sensillum shaft compartmentalizing sensory lymph bathing the neuronal dendrites (Shanbhag et al., 2000). Olfactory sensilla are lined with pores open to the external environment and function as isolated microenvironments maintained by multiple cell types wherein sensory neurons receive and transduce odor information (Steinbrecht, 1996; Ando et al., 2019). Collectively, odor information is encoded through varying degrees of combinatorial activation of specific odor-sensitive OSNs both within and across sensilla, which allows a finite number of receptors to encode an extensive variety of odor information present in the environment (Grabe and Sachse, 2017; Seki et al., 2017; Haverkamp et al., 2018).

To dynamically and sensitively discriminate odors, ORs evolved in the terrestrial context of a challenging and turbulent odorscape of flight, wherein odor information is temporally and spatially intermittent, noisy, and dynamically varying (Koehl, 2006; Brand et al., 2018). Insect ORs are distinct from vertebrate olfactory receptors, in that they form heteromeric transmembrane ion channels capable of conducting cation inflow and possess an inverted topology (intracellular N- and extracellular C-termini) unlike vertebrate ORs which are a subset of conventional G protein-coupled receptors (GPCRs) with extracellular N-termini and intracellular C-termini (Benton et al., 2006; Lundin et al., 2007). Insect ORs form heteromers consisting of an odor-binding OR and a highly conserved odorant receptor co-receptor (Orco). Orco is required for the correct trafficking, localization, and functioning of OR complexes (Larsson et al., 2004; Benton et al., 2006), and is a necessary component of the complex for odorant sensitivity (Larsson et al., 2004). Cryo-electron microscopic studies have shed further light on the structural basis of insect OR complexes: in the absence of odor-tuning ORs, Orco isolated from the parasitic fig wasp *Apocrypta bakeri* alone forms homotetramers (Butterwick et al., 2018). Similarly, in the jumping bristletail *Machilis hrabei*, a basal insect that does not encode the olfactory co-receptor Orco in its genome, though is considered to harbor ancestral members of the OR family, a single OR seems to also form homotetramers with broad ligand tuning (del Mármol et al., 2021). Due to partial sequence conservation among Orco and tuning ORs, a similar tetrameric channel architecture with a central cation-conducting pore is thought to occur *in vivo*, in native, heteromeric, OR complexes that feature ORs and Orco (Butterwick et al., 2018). OR complexes are thus composed of co-receptor and odor-tuning receptor channels, wherein odorants rapidly and directly elicit cation influx to subsequently depolarize and activate OSNs.

Crucially, in addition to this fast and primary “ionotropic” mode of action, insect OR complexes can also act as elicitors of intracellular, “metabotropic” signaling through the activation of secondary messengers which can further modulate the OR complex and the transduction event, akin to vertebrate ORs (Krieger and Breer, 1999; Wicher, 2015). Vertebrate ORs are receptors that couple through a G protein cascade to a secondary messenger pathway, which opens cyclic nucleotide-gated cation channels to enact sensory transduction responses (Pace et al., 1985; Nakamura and Gold, 1987; Zufall et al., 1994; Bazáes et al., 2013; Zufall and Domingos, 2018). Indeed, insect ORs were first conceptualized as mere metabotropic receptors, following the G protein-coupling schema of vertebrate ORs known at the time (Wetzel et al., 2001; Zufall and Domingos, 2018). In subsequent years, various G proteins were discovered to be expressed in *Drosophila* antennae (Boto et al., 2010) and have been shown to play roles in olfactory responses in OSNs (Kain et al., 2008; Deng et al., 2011; Ignatious Raja et al., 2014). Downstreaming the cascade, the function of the cyclic nucleotide cAMP has been linked to *Drosophila* olfactory performance. Experimental increases in intracellular cAMP in OSNs result in elevations in neuronal resting activity and OSN excitation (Deng et al., 2011) and render OSNs more sensitive at lower odorant concentrations (Getahun et al., 2013). Conversely, exogenous reductions in cAMP level result in diminished responses to odor stimulations (Gomez-Diaz et al., 2004; Getahun et al., 2013). When heterologously expressed in HEK293 cell lines, insect OR complexes have been shown to open and conduct ionic currents in response to both cyclic nucleotides cAMP and cGMP (Wicher et al., 2008), common intracellular effectors general and ubiquitous to eukaryotic life. Orco phosphorylation by protein kinase C (PKC) is a prerequisite of cAMP activation of ORs in sensillum microinjection experiments (Sargsyan et al., 2011). In parallel, cAMP production in OSNs is stimulated by odor-induced OR activation and can proceed in a manner dependent or independent of intracellular Ca^2+^ (Miazzi et al., 2016).

Furthermore, the important sensory and perceptual properties of neuronal sensitization and de-sensitization (adaptation) in the context of repeated odor stimulation are thought to be underpinned by metabotropic activity within OSNs (Murmu et al., 2011; Guo et al., 2017; Jafari and Alenius, 2021). For instance, repeated sub-detection threshold of odor stimulations produces stronger responses in the antennal sensilla of live flies (Getahun et al., 2013), as well as in a CHO cell line expressing *D. melanogaster* ORs (Mukunda et al., 2016). The capacity to sensitize is related to intracellular Ca^2+^ dynamics and Ca^2+^ buffering and storage; for instance, sensitization is abolished upon inhibition of calmodulin (CaM) (Mukunda et al., 2016). Although it is not clear whether OSN sensitization is a general feature among all OSN subtypes, recent evidence indicates that some functional heterogeneity exists between OSNs, which may depend on OR subunit or OSN subtype (Halty-deLeon et al., 2021). Inversely, the property of sensory adaptation is also evident in OSNs: decreases in spike amplitude upon prolonged odor stimulation in single-sensillum recordings are widely observed and are proportional to stimulus intensity and duration (Zufall and Leinders-Zufall, 2000; Martin and Alcorta, 2016; Guo and Smith, 2017; Gomez-Diaz et al., 2018). The odorant presentation also induces dephosphorylation of serine residues on the Orco coreceptor, which is linked to partial but not complete receptor desensitization (Guo and Smith, 2017; Guo et al., 2017). Metabotropic modes of regulating odor transduction by way of coupled secondary pathways in insect OSNs, therefore, allow these sensory systems to tune their responses in ecologically relevant ways already at the sensory and receptor levels.

However, striking exceptions to this “merely regulatory” rule for metabotropy exist. In the exquisitely sensitive detection of sex pheromones in trichoid sensilla of the hawkmoth *Manduca sexta*, ORs seem to function exclusively via metabotropic means, with no apparent evidence for ionotropic transduction *in vivo* (Stengl, 2010; Nolte et al., 2013, 2016). Transduction is instead reliant on G protein-dependent phospholipase C (PLC)-dependent transient receptor potential (TRP)-like channels, which act as the primary transducers of pheromone odorants (Gawalek and Stengl, 2018). Based on follow-up studies employing ion current measurements in trichoid sensilla, sensitization and adaptation mechanisms in pheromone sensilla were indeed later shown to be cAMP- and cGMP-dependent, respectively (Dolzer et al., 2021). Multiple disparate lines of evidence, therefore, implicate cyclic nucleotides and coupled signaling cascades as important elements in odor transduction and regulators of OR performance events across insects; these and other modes of OR regulation beyond the present scope have been extensively reviewed (Fleischer et al., 2018; Wicher, 2018; Wicher and Miazzi, 2021).

One unexplored, potential mode of signaling in insect antennae that utilizes the cyclic nucleotide cGMP is that of nitric oxide (NO) signaling. NO is a gaseous, short-range, diffusion-limited signal and has since been well-documented as a messenger mediating diverse signaling pathways within and across cells (Garthwaite, 2008; Friebe and Koesling, 2009; Steinert et al., 2010, 2011). NO is produced endogenously by a Ca^2+^/CaM-sensitive nitric oxide synthase (NOS) and rapidly diffuses in a cell membrane-permeable manner as an isotropic paracrine or autocrine messenger (Regulski and Tully, 1995; Tuteja et al., 2004; Bryan et al., 2009). NO then binds to its chief receptors, the NO-sensitive soluble guanyl cyclases (sGC), which catalyze an intracellular production of cGMP from its precursor, GTP (Denninger and Marletta, 1999; Derbyshire and Marletta, 2012). cGMP thus accumulates and is the terminal effector of the NO-cGMP signaling cascade. Canonically, cGMP is known to widely act on three receptor classes: cGMP-dependent protein kinases (Tuteja et al., 2004; Bryan et al., 2009), cGMP phosphodiesterases, through which cGMP is removed via hydrolysis (Maurice et al., 2014), and cyclic nucleotide-gated channels (Biel et al., 1998), which are potentially crucial effectors of signal transduction in sensory neurons (Kaupp and Seifert, 2002; Pifferi et al., 2006; Kaupp, 2010).

To date, NO has been described and implicated as an active physiological modulator in various sensory systems characterized by sensory lymph alike to those found in insect chemosensory sensilla. For example, NOS activity and NO signaling have been well-documented in the mammalian cochlea, the lymphatic site of auditory transduction (Fessenden et al., 1994; Fessenden and Schacht, 1998; Kopp-Scheinpflug and Forsythe, 2021). NO signaling is also evident in olfactory transduction. The involvement of cyclic nucleotides is observable in cultured rat OSNs where cGMP production by sGC is NO-dependent and increases cytosolic [Ca^2+^] (Schmachtenberg et al., 2003; Pietrobon et al., 2011). Adult mouse OSNs liberate NO in an odor-dependent fashion and contribute to sensory adaptation (Brunert et al., 2009). Transcriptomic atlases of the mouse olfactory mucosa also show high sGC transcript enrichment in specific zones of the tissue (Ruiz Tejada Segura et al., 2022). NOS presence and activity are also evident in mature OSNs of *Caudiverbera* and *Xenopus* frogs (Schmachtenberg and Bacigalupo, 1999, 2000), as well as in the larvae of sea lampreys, the most basal group of vertebrates, where NOS is present in OSNs and other cells of the olfactory periphery, such as sustentacular and basal cells (Zielinski et al., 1996). Furthermore, chemosensing mechanisms in *C. elegans* and mammals that do not involve GPCR-mediated transduction, but rather cGMP-dependent detection and signaling mechanisms that utilize both soluble and membrane receptor guanyl cyclases, also exist (Bargmann, 2006; Leinders-Zufall et al., 2007; Bleymehl et al., 2016). NOS has also been described in the chemosensory neurons of diverse invertebrates such as cuttlefish (Scaros et al., 2018), gastropods (Elphick et al., 1995a; Wyeth and Croll, 2011), and in the hydra, a primitive invertebrate harboring the simplest form of an olfactory system across multicellular life, where it is involved in the feeding response (Colasanti et al., 1995). Indeed, it seems that OSN-specific expression of NOS and OSN sensitivity to NO is a broadly documented characteristic among non-insect animals.

In insects specifically, NO-cGMP signaling is known to function in sensory systems (Elphick et al., 1993; Elphick and Jones, 1998; Davies, 2000; Bicker, 2001; Orr et al., 2001) and the relevance of this signaling pathway across a breadth of neur(on)al processes has been reviewed (Wright, 2019). Multiple precedents exist for the involvement of NO-cGMP modulation specifically in insect sensory systems. NO signaling is described in the locust visual system, where NO and cyclic GMP modulate photoreceptor cell responses (Schmachtenberg and Bicker, 1999; Orr et al., 2001). This partly parallels NO involvement in vertebrate retinas, where light-dependent NO production activates sGC and resultant cGMP elevation mediates ionic conductance in the retinal rods, cones, bipolar and ganglion cells, as well as affects local gap junction coupling (Vielma et al., 2012). More yet, and specifically in the context of insect olfaction, NO signaling has been found in antennal lobes, the primary olfactory processing center of insect brains, where NOS localization studies have revealed NOS and sGC expressions in adult moths (Nighorn et al., 1998). Subsequent research identified complementary expression of NOS and SGCs in mutually exclusive cell populations in antennal lobes and demonstrated weakening of the NO signal as determined by functional fluorescence imaging employing a NO-sensitive dye during pharmacological inhibition of NOS (Collmann et al., 2004). NO signaling has also been found to play a requisite role in the correct olfactory perception and learning of honeybees (Müller and Hildebrandt, 1995, 2002; Menzel and Müller, 1996; Müller, 1996; Hosler et al., 2000; Dacher and Gauthier, 2008), locusts (Elphick et al., 1995b, 1996), and moths (Higgins et al., 2012; Gage et al., 2013). Across these investigations in insects, experiments involving NO signaling pathway manipulations and discoveries of co-localization of NO production and reception have thus solidified NO as an important messenger in olfactory processing in insect brains.

However, NO signaling has not been investigated in the sensory olfactory periphery of insects, i.e., at the antennal level (Davies, 2000; Wright, 2019). In fact, to our knowledge, only two indirect and circumstantial references exist in this regard: NOS expression is differentially expressed between antennae of drone and worker honey bees (Jain and Brockmann, 2020), and NOS gene transcripts are highly expressed in antennal tissue samples of the *M. sexta* hawkmoth as surveyed by Northern blot and RT-PCR (Nighorn et al., 1998). Given previous evidence that cGMP can directly activate Orco channels (Wicher et al., 2008) and that cGMP is involved in olfactory transduction in insect model systems (Flecke et al., 2006; Dolzer et al., 2021), we opted to explore the NO signaling pathway as a mode of signaling in antennae. In particular, we ask whether NO acts as a functional messenger involved in the regulation and potentiation of olfactory responses in live insect antennae, as is evident in non-insect olfactory sensory systems as well as insect non-olfactory sensory systems. As a prediction, we thus hypothesize that the NO-cGMP pathway plays a role in potentiating olfactory responses in insects. Here, we pay special attention to recapitulate a native, *in vivo* context of antennal functioning, given that much of the preceding work has focused on ectopic expression of ORs in simple *in vitro* heterologous expression systems and non-native environments such as “empty neuron” systems (Dobritsa et al., 2003; Kurtovic et al., 2007).

In this study, we first consulted bioinformatic resources and queried various antennal tissue transcriptomes for the expression of pathway-relevant signaling genes in *D. melanogaster*. Subsequently, we used real-time optical and electrophysiological recordings in live *D. melanogaster* antennae in the presence and absence of modulators of both NO and cyclic nucleotide pathways to functionally test for sensory response effects. The pharmacological aspect is enabled by previous work characterizing *Drosophila* NOS and NO receptors, the soluble guanyl cyclases, using a wide panel of pharmacological tools capable of inhibiting and activating the entire NO-cGMP pathway, either through the provision of NO itself or by modulating resultant cGMP activity (Nighorn et al., 1999; Langlais et al., 2004; Morton, 2004; Morton et al., 2005). Finally, we also perform experiments comparing response effects between cGMP and cAMP, using both long- and short-time-separated odor stimulation experiments, as both cyclic nucleotides have been suggested to exert opposite effects on OSN responses *in vivo* as crucial elements of metabotropic transduction cascades (Flecke et al., 2006; Dolzer et al., 2021). Contrary to expectation, we find no response effect during modulation of the NO-cGMP pathway and find no effect of cGMP application on responses using both calcium imaging of OSNs as well as single sensillum recordings. The result stands in contrast to cAMP, for which a response effect was evident, but which is not a downstream element of the NO signaling cascade. We speculate whether NO may instead function beyond the antenna or in a non-olfactory capacity at the antennal level as a paracrine signal among the heterogeneous cell types of the antenna, of which some co-activate upon odor stimulation (Prelic et al., 2022; Calvin-Cejudo et al., 2023).

## 2. Material and methods

### 2.1. Transcriptomic analysis

Three independent RNA-seq studies with antennal tissue-derived transcriptomes were used to ascertain antennal gene expression with cross-study robustness. We consulted an RNA-seq study pooling 300 mixed-sex flies, 5–12 days old post-eclosion, for two *D. melanogaster* genotypes: wildtype Canton-S flies and atonal (ato) mutants (Menuz et al., 2014). This study was selected to provide hints about local olfactory subsystem expression. Another RNA-seq dataset sampling 1,200 antennae pairs for each sex in Canton-S flies aged >1-day post-eclosion was also consulted to compare between flies sex (Shiao et al., 2013). Finally, we also consulted a set of transcriptomes comparing antennal expression in six *Drosophila spp*. wherein 300 mixed-sex antennae for each species in flies aged 7–10 days post-eclosion were sampled (Pan et al., 2017).

Genes to consider were selected based on known involvement in the nitric oxide signaling pathway, or as annotated with Gene Ontology (GO) annotations involving nitric-oxide synthase activity (GO:0004517), cGMP-dependent protein kinase activity (GO:0004692), guanylate cyclase activity (GO:0004383), cGMP biosynthetic process (GO:0006182), cGMP binding (GO:0030553), or intracellular cGMP-activated cation channel activity (GO:0005223). Shortlisted genes functionally span the whole hitherto known NO-cGMP cascade involving the production and elicitors of the NO signal. The gene list includes Nos (*Drosophila* nitric oxide synthase); Gycα99B and Gycβ100B (nitric oxide-sensitive soluble guanyl cyclases); Gyc88E, Gyc89Da, and Gyc89Db (atypical soluble guanyl cyclases); Gyc32E, Gyc76C, CG42637, CG34357, CG33958, CG31183, CG10738, and CG3216 (membrane-associated guanyl cyclase receptors uninvolved in NO reception); for, PkG21D, and CG4839 (cGMP-dependent protein kinases); CngA, CngB, Cngl, and CG42260 (cGMP-gated ion channels); and finally, Pde1c, Pde6, Pde9, and Pde11 (cGMP phosphodiesterases). We also selected several highly expressed antennal control genes involved in olfactory reception and transduction processes: the olfactory (co)receptors Orco, Or67d, and Ir8a, as well as common pancellular housekeeping genes Act5C, Cam, and Gapdh1, involved ubiquitously as cytoskeleton component, messenger protein, and metabolic protein, respectively. All gene nomenclature presented herein is based on FlyBase's (flybase.org) gene symbol and name; in studies incorporating outdated names or Flybase ID, gene labels were converted to gene FlyBase symbol and name (e.g., where applicable, Or83b was renamed to Orco for cross-study consistency).

A gene's expression percentile ranking was determined by identifying the rank of a gene's average expression in a subset of the data containing non-zero-expressed genes, set as a percentage of the dataset; for this, the PERCENTRANK function in Excel was used on an array of data excluding genes not detected in antennal transcriptomes (i.e., where RPKM average or FPKM average = 0).

All single-cell transcriptomes used were obtained from the Fly Cell Atlas (Li et al., 2022). The datasets used come from antennal tissue-specific scRNA-seq with cells isolated either using a microfluidic droplet-based cell-capture 10X methodology or the plate-based SMART-seq2 methodology (Li et al., 2017, 2022; McLaughlin et al., 2021). Both 10X datasets originating from “stringent” and “relaxed” datasets were data mined in parallel with the SMART-seq2-derived dataset. Cell group classifications are based on the groupings “*annotation_broad*” and “*annotation*” for all 10X antennal datasets, and “*transf_annotation*” for the SMART-seq2-derived antennal dataset. Datasets merging 10X- and SMART-seq2-sourced transcriptomes were not considered to avoid confoundment.

The single-cell transcriptomic visualization platform SCope (Davie et al., 2018) was used for visualizing tSNE plots where gene expression (corrected transcript count) is visualized by color on a min-max basis using default settings.

All differential expression analyses performed comparing two cell types on specific gene expression used the non-parametric Wilcoxon signed-rank statistical test (via Seurat) with default parametrization using the online Automated Single-cell Analysis Pipeline ASAP portal at asap.epfl.ch (Gardeux et al., 2017). The parameters used were as follows: *minimum % of cells with gene* > *0* = 0.1 (10%); *false detection rate limit* = 0.05; *min%diff* = NULL; *max cells per group* = NULL; *Foldchange cutoff* = 1.3; and Max cells per group = NULL. *Foldchange cutoff* was set to 1.3 or 2.0 (i.e., ~2.5- and 4-fold difference in expression) to differentiate between weak and strong detection of gene transcript enrichment or depletion between cell groups. Differentially expressed genes detected at *Foldchange cutoff* = 1.3 but not at 2.0 were shaded in light color, while genes detected at 2.0 but not below were shaded in dark color. Additionally, genes detected as significantly upregulated, undetected as neither enriched nor depleted, or downregulated in a specific cell category compared to the complementary set (i.e., all other antennal cells) were shaded in green, gray, or red, respectively. Here, as before, a variety of queried antennal datasets from the Fly Cell Atlas were selected for comparative robustness. We looked at data originating from different single-cell isolation methods (10X and SMART-seq2), within datasets generated from raw data by different data processing pipelines (*stringent* vs. *relaxed* datasets), and across different kinds of annotations of cell type, which are categorized manually by crowd annotation or through clustering (e.g., “*annotation_broad*” discriminates broadly between general cell type; “*annotation”* discriminates between cell subtype, especially within the sensory neuron class).

### 2.2. Fly lines and rearing

*D. melanogaster* fly line expressing the fluorescent “fast kinetic” cytoplasmic free-Ca^2+^ indicator GCaMP6f (Chen et al., 2013) under UAS control was obtained from Bloomington Drosophila Stock Center, Indiana (bdsc.indiana.edu), stock number 42747. The line was crossed with an Orco-Gal4 line to produce a stable line expressing GCaMP6f in Orco^+^ cells (OSNs), of genotype +;UAS-GCaMP6f/(CyO);Orco-Gal4/(TM6B), which has been validated and used in previous studies (Mukunda et al., 2014, 2016; Miazzi et al., 2016; Halty-deLeon et al., 2018, 2021; Prelic et al., 2022). Fly stocks used for microinjection experiments are detailed below. Flies were maintained on conventional cornmeal agar medium (recipe available in a data repository) in incubation under a 12-h/12-h light/dark cycle at 25°C and 70% humidity. The study was conducted in Germany where research on invertebrates requires no animal research committee approval. The transgenic fly laboratory meets all requirements of the Thuringian State Office for Consumer Protection (verbraucherschutz.thueringen.de).

### 2.3. Open antenna preparation

Antennae of 1–18-day-old flies were excised and prepared as described previously (Mukunda et al., 2014; Halty-deLeon et al., 2018; Prelic et al., 2022). In brief, flies were anesthetized on ice; antennae were then excised using a fine needle, and deposited into a 100 ml droplet of *Drosophila* Ringer solution (5 mM HEPES; 130 mM NaCl; 5 mM KCl; 2 mM MgCl_2_; 2 mM CaCl_2_; 36 mM sucrose) equilibrated before pH = 7.30 and room temperature. Excised antennae were then fixed into a vertical position on a coverslip with a two-component silicone curing gel (KWIK-SIL, World Precision Instruments, wpi-europe.com). Thereafter, the antennal preparation was immersed in 100 ml Ringer solution to maintain tissue tonicity and prevent drying, and cut horizontally with micro-scissors to expose a layer of antennal tissue for immediate imaging. Antennae remained immersed for the duration of experiments and a maximum of 30 min. All experiments were carried out during the day (light cycle). Before use, all Ringer solutions were equilibrated to room temperature and pH = 7.30.

### 2.4. *Ex vivo* calcium imaging

Ca^2+^ imaging of antennae originating from flies expressing the cytoplasmic free-Ca^2+^ sensor GCaMP6f in an OSN-restricted manner (driven by Orco-Gal4) was performed with an epifluorescence microscope (Axioskop FS, Zeiss, Jena, Germany) coupled to a monochromator (Polychrome V, Till Photonics, Munich, Germany). A water immersion objective (LUMPFL 40 × W/IR/0.8; Olympus, Hamburg, Germany) was used along with an imaging control unit (ICU, Till Photonics). A 490 nm dichroic mirror and a 515 nm long-pass filter were employed to filter emitted light for capture with a cooled CCD camera controlled by TILLVision 4.5.62 software (TILL Photonics). An experimental protocol was programmed to sample images every 5 s over 180–250 imaging cycles, allowing for continuous specimen imaging. Each sampling event follows a 50 ms exposure to 475 nm light generated by the monochromator.

In all experiments, antennae prepared on coverslips were placed into a custom-made flow chamber (1.5 ml volume) which was used to provide the continuous laminar flow of a bath solution (~1 ml min^−1^) across the imaged antenna. All chemical applications to the sample were performed by manually pipetting a stimulant solution of 50 μl volume onto the immersed objective at 45° incidence, for advection and diffusion over the submerged antennal cross-section being imaged. In most cases, the first VUAA1 pulse was pipetted during imaging cycle 30. The bath solution was exchanged at cycle 50 (with the new solution first making contact with the bath chamber at cycle 66) or for “short exposure” experiments at cycle 100 (solution making contact with the chamber at cycle 116). Second VUAA1 stimulations were co-applied with a pharmacological agent during imaging cycle 130 in all cases. After recording completion (imaging cycle 220), a background region beyond the antennal edge was marked along with observed GCaMP6f-labeled OSNs, which were marked as regions of interest (ROIs). TILLVision software was used to generate a matrix of average fluorescence values for the background region and all ROIs; this matrix was exported for data analysis using *R*. All raw and processed data are available in the data repository indicated in the Data availability statement Section.

### 2.5. Data analysis and visualization

Cation imaging response magnitudes were calculated as average changes in ROI fluorescence signal subtracted from background signal, relative to a non-response baseline over 10 imaging cycles (50 s) preceding the first stimulation, and so converted into percentage change relative to baseline (ΔF/F_0_), as used previously (Mukunda et al., 2014; Halty-deLeon et al., 2018; Prelic et al., 2022). A custom script was written in *R* to transform the exported matrix of raw fluorescence intensity values into ΔF/F_0_ time course plots for each of the regions of interest as marked on the open antenna. The script reads a batch of replicates to produce a final time course plot showing all replicates and an average with its standard error of the mean (SEM). First, background noise is subtracted from all ROIs in each antenna (replicate) for background noise correction. Second, each time course is normalized to a baseline of 0 based on a common “resting” F_0_ time window (10 imaging cycles over 50 s, during imaging cycles 20–29) before the first stimulation, so that biological replicates can be compared. Finally, a mean average and SEM are calculated for each time point across all replicates (individual antennae) or ROIs (not shown; available in a data repository). The script produces two outputs: a table of processed data (for purposes of statistical analysis) and a time course graph. Here, the calculated average time course is plotted superimposed on its source replicates to show both individual and the grouped average trend, along with labels demarcating treatment time points, and a gray-shaded interval showing the time window used for normalizing each ROI recording (F_0_ time window). For bar charts comparing baseline and peak response levels, the time point 5 s before stimulation is always used as the baseline, and the time point at which a local maximum in average response across antennae is reached was used as the peak response point. The non-parametric two-tailed Wilcoxon matched-pairs signed rank testing was used to compare response peaks, given that some response peaks did not follow a Gaussian distribution as tested by the Shapiro–Wilk test for normality (not shown). The Wilcoxon test was also selected as a more conservative test approach in lieu of the fact that live cell fluorescence was measured across a heterogeneous population of OSNs (driven by the broad OSN-targeting Orco-Gal4) appearing in- as well as closely out-of-plane of imaging, further adding measurement variation. For bar charts reporting time-to-response-peak values, averages for all ROIs across antennal replicates were used. All error bars represent SEM and corresponding peak response latencies are reported in parentheses on x-axis labels. The non-parametric Kruskal–Wallis test with Dunn's multiple comparisons test was used to compare time-to-response-peak values across experiments. Statistical comparisons are also detailed in figure legends wherever statistical tests are performed; n.s. denotes statistical insignificance (*p* > 0.05) and asterisks indicate statistical significances, which are explicated in-text if present. Statistical analyses were performed using GraphPad Prism 9 (graphpad.com), Rstudio (rstudio.com), and Microsoft Excel. All raw and processed data, as well as analyses not mentioned in this study which were performed on an ROI-by-ROI basis rather than an antenna-by-antenna basis, are available in the data repository indicated in the Data availability statement Section.

### 2.6. Electrophysiology (single sensillum recording and microinjections)

Electrophysiological single sensillum recordings and microinjections were performed on *D. melanogaster* flies of genotype Or22a-GAL4;UAS-mCD8-GFP, expressing membrane-tagged GFP in Or22a-expressing ab3A OSNs, as performed previously (Olsson et al., 2011; Getahun et al., 2013). In short, 2–5-day-old adults were fixed dorsally to a microscope slide. For odor stimulation, 10 μl of 10^−5^ ethyl butyrate (Sigma, Taufkirchen, Germany) dissolved in hexane (99%, Fluka Analytical, Buchs, Switzerland) was pipetted onto 1 cm filter paper using a disposable Pasteur pipette. Charcoal-filtered and humidified air (~1 l min^−1^) passed over the antenna from a stimulus air controller (Syntech, CS-5, Hilversum, NL) through an aluminum tube ~10 mm from the antenna. During stimulation, airflow bypassed a complementary air stream (0.5 l/min during 0.5 s) through the stimulus pipette placed roughly 3 cm from the preparation. Compounds and concentrations for injection were diluted in saline (Kaissling and Thorson, 1980; Olsson et al., 2011) as follows: 8-bromo-cAMP (1 mM) and 8-bromo-cGMP (1 mM). Prepared concentrations of injected agents were 100-fold higher than the concentration used in previously isolated cell preparations due to a dilution effect in sensilla (Olsson et al., 2011). To check whether any injected compounds reached OSN outer dendrites, we injected the Or22a agonist ethyl butyrate at threshold concentration (−9 v/v) into the base of ab3 sensilla as a diagnostic test. During a 200-s injection period, ethyl butyrate enhances the activity of the ab3A neuron expressing Or22a responding to air stimulations of ethyl butyrate, and no change in activity is observed for the ab3B neuron (Getahun et al., 2013), thus excluding non-specific effects. We also excluded mechanical artifacts that could affect OSN activity during long-lasting injection; effects of microinjecting saline and modulators dissolved therein were determined not to change OSN spontaneous (“resting”) activity over the 300 s recording period (Getahun et al., 2013).

Responses were then analyzed between 500 and 1,350 ms after stimulus onset, accounting for mechanical stimulus delay (150 ms). For response kinetics, spike frequency ratios were analyzed as peri-stimulus time histograms (PSTHs) in 25 ms bins by dividing each 25 ms frequency by the average pre-stimulus frequency over 2 s to give a normalized ratio for each time point. The PSTHs presented in the figures show normalized means ± standard error of mean for n cells. Areas under the PSTH curve (AUC) were measured for each response profile using the trapezoid rule, using GraphPad Prism 9, and divided by the time to establish a normalized frequency average for each response. A total of 10, 11, 12, and 10 replicates (fly individuals) were performed for ethyl butyrate response recordings in the no microinjection control group, a saline microinjection control group, 8-bromo-cAMP, and 8-bromo-cGMP microinjection groups, respectively.

### 2.7. Chemicals

VUAA1 (N-(4-ethylphenyl)-2-((4-ethyl-5-(3-pyridinyl)-4H-1,2,4-triazol-3-415 yl)thio)acetamide) was synthesized by the Mass Spectrometry/Proteomics group at the Max Planck Institute for Chemical Ecology (Jena, Germany). VUAA1 was dissolved in dimethyl sulfoxide (DMSO) (Sigma) to yield a 100 mM stock solution. Dissolved VUAA1 stocks were stored for a maximum of 3 weeks at −20°C. Before each experiment, the stock solution was dissolved 1:5,000 in freshly equilibrated (pH = 7.30) *Drosophila* Ringer solution to yield fresh 20 μM VUAA1 application solutions to be used as an odor proxy stimulant during Ca^2+^ imaging experiments.

ODQ (1H-[1,2,4]oxadiazolo[4,3-a]quinoxalin-1-one) (Cat. No. 0880, Tocris Bioscience) was dissolved in DMSO to yield a 10 mM stock solution. ODQ stocks were stored for a maximum of 3 days and stored at −20°C. Before each experiment, the stock solution was dissolved 1:1,000 in freshly equilibrated (pH = 7.30) *Drosophila* Ringer solution to yield a fresh 10 μM ODQ bath solution which was used to perfuse antennae during Ca^2+^ imaging and act as a solvent for VUAA1 applications in the presence of Ringer solution with 10 μM ODQ.

SNP was prepared from sodium nitroprusside dihydrate (CAS: 13755-38-9, Prod. No. 71780, Fluka Analytical, Honeywell). Sodium nitroprusside dihydrate was dissolved in water to yield a 10 mM stock solution. SNP stocks were stored for a maximum of 7 days at −20°C. Before each experiment, the stock solution was dissolved 1:1,000 in freshly equilibrated (pH = 7.30) *Drosophila* Ringer solution to yield a fresh 10 μM SNP bath solution which was used to perfuse antennae during Ca^2+^ imaging and act as a solvent for VUAA1 applications in the presence of Ringer solution with 10 μM SNP.

L-NAME (N(gamma)-nitro-L-arginine methyl ester hydrochloride) (Cat. No. 0665, Tocris Bioscience) was dissolved to prepare a 10 mM L-NAME stock in *Drosophila* Ringer solution. L-NAME stocks were stored for a maximum of 7 days at −20°C. Before each experiment, the stock solution was dissolved 1:1,000 in freshly equilibrated (pH = 7.30) *Drosophila* Ringer solution to yield a fresh 10 μM L-NAME bath solution which was used to perfuse antennae during Ca^2+^ imaging and act as a solvent for VUAA1 applications in the presence of Ringer solution with 10 μM L-NAME.

8Br-cGMP (8-Bromoguanosine 3′,5′-cyclic monophosphate sodium salt) (CAS: 51116-01-9, Prod. No. 203820, Calbiochem, Sigma-Aldrich) was dissolved to prepare a 10 mM 8Br-cGMP stock in distilled water. 8Br-cGMP stocks were stored for a maximum of 7 days at −20°C. Before each experiment, the stock solution was dissolved 1:1,000 in freshly equilibrated (pH = 7.30) *Drosophila* Ringer solution to yield a fresh 10 μM 8Br-cGMP bath solution which was used to perfuse antennae during Ca^2+^ imaging and act as a solvent for VUAA1 applications in the presence of Ringer solution with 10 μM 8Br-cGMP.

8Br-cAMP (8-Bromoadenosine 3′,5′-cyclic monophosphate sodium salt) (CAS: 76939-46-3, Prod. No. B7880, Sigma-Aldrich) was dissolved to prepare a 10 mM 8Br-cAMP stock in distilled water. 8Br-cAMP stocks were stored for a maximum of 7 days at −20°C. Before each experiment, the stock solution was dissolved 1:1,000 in freshly equilibrated (pH = 7.30) *Drosophila* Ringer solution to yield a fresh 10 μM 8Br-cAMP bath solution which was used to perfuse antennae during Ca^2+^ imaging and act as a solvent for VUAA1 applications in the presence of Ringer solution with 10 μM 8Br-cAMP.

Forskolin [(3*R*,4a*R*,5*S*,6*S*,6a*S*,10*S*,10a*R*,10b*S*)-3-Ethenyl-6,10,10b-trihydroxy-3,4a,7,7,10a-pentamethyl-1-oxododecahydro-1*H*-naphtho[2,1-*b*]pyran-5-yl acetate] (CAS: 66575-29-9, Prod. No. F6886, Sigma-Aldrich) was dissolved in DMSO to make a 10 mM stock solution (Huang et al., 1982). Stocks were stored for a maximum of 7 days at −20°C. Before each experiment, the stock solution was dissolved 1:1,000 in freshly equilibrated (pH = 7.30) *Drosophila* Ringer solution to yield a fresh 10 μM forskolin bath solution which was used to perfuse antennae during Ca^2+^ imaging and act as a solvent for VUAA1 applications in the presence of Ringer solution with 10 μM forskolin.

## 3. Results

### 3.1. NO signaling pathway genes are expressed in OSNs in drosophilid antennae

To test the putative role of NO signaling in long-term regulation in *Drosophila* olfactory tissues, we asked whether genes involved in the NO signaling pathway are expressed in the *Drosophila* antenna. For this, we surveyed a variety of antennal tissue-specific and single-cell antennal transcriptomes available, and queried these datasets for expression abundance of genes involved in various stages of the nitric oxide signaling pathway, related enzymes, and control genes, to gauge whether NO signaling machinery is present in the antenna of adult flies. Specifically, we selected several genes involved as core participants in the NO signaling pathway (*Drosophila* nitric oxide synthase Nos, and both conventional NO-sensitive soluble guanyl cyclases Gycα99B and Gycβ100B), related but pathway-uninvolved genes such as constituents of atypical soluble guanyl cyclase heterodimers (NO-insensitive guanyl cyclases Gyc88E, Gyc89Da, Gyc89Db) and membrane-associated guanyl cyclase receptors (Gyc32E, Gyc76C, CG42637, CG34357, CG33958, CG31183, CG10738, CG3216), as well as effectors or genes known for their involvement as targets of the NO signaling pathway: the cGMP-dependent protein kinases (for, Pkg21D, and CG4839), cGMP-gated ion channels (CngA, CngB, Cngl, and CG42260), and cGMP phosphodiesterases (Pde1c, Pde6, Pde9, and Pde11). For comparison, we selected six control genes: the OR and IR olfactory subsystem co-receptors Orco and Ir8a, the fly pheromone cis-vaccenyl acetate receptor Or67d, and three common highly expressed housekeeping genes, Act5C, Cam, and Gapdh1. The motivation for the choice and involvement of this comprehensive list of genes is summarized in Table 1.

**Table 1:** Gene panel selected for involvement in the *Drosophila* nitric oxide signaling pathway.

**Gene symbol**	**Full gene name**	**NO signaling pathway involvement**	**Function (localization)**	**Exemplary references**
Nos	Nitric oxide synthase	Direct (signal production)	Production of nitric oxide signal	Regulski and Tully, 1995; Stasiv et al., 2001
Gycalpha99B	Guanylyl cyclase α-subunit at 99B	Direct (signal reception)	NO receptor, soluble guanyl cyclase	Liu et al., 1995; Shah and Hyde, 1995; Morton et al., 2005
Gycbeta100B	Guanylyl cyclase β-subunit at 100B	Direct (signal reception)	NO receptor, soluble guanyl cyclase	Shah and Hyde, 1995; Morton et al., 2005
Gyc88E	Guanylyl cyclase at 88E	None	Constituent of atypical soluble guanyl cyclase heterodimers; NO-insensitive oxygen sensor	Langlais et al., 2004; Morton, 2004; Morton et al., 2005; Huang et al., 2007; Luo et al., 2009
Gyc89Da	Guanylyl cyclase at 89Da	None	Constituent of atypical soluble guanyl cyclase heterodimers; NO-insensitive oxygen sensor	Morton, 2004; Morton et al., 2005; Vermehren-Schmaedick et al., 2010
Gyc89Db	Guanylyl cyclase at 89Db	None	Constituent of atypical soluble guanyl cyclase heterodimers; NO-insensitive oxygen sensor	Langlais et al., 2004; Morton, 2004; Morton et al., 2005; Vermehren-Schmaedick et al., 2010
Gyc32E	Guanylyl cyclase at 32E	None	Inferred membrane-associated guanyl cyclase receptor	Gigliotti et al., 1993
Gyc76C	Guanylyl cyclase at 76C	None	Membrane-associated guanyl cyclase receptor	Liu et al., 1995; Overend et al., 2012; Chak and Kolodkin, 2014; Schleede and Blair, 2015
CG42637	–	None	Inferred membrane-associated guanyl cyclase receptor	FlyBase
CG34357	–	None	Inferred membrane-associated guanyl cyclase receptor	FlyBase
CG33958	–	None	Inferred membrane-associated guanyl cyclase receptor	FlyBase
CG31183	–	None	Inferred membrane-associated guanyl cyclase receptor	FlyBase
CG10738	–	None	Inferred membrane-associated guanyl cyclase receptor	FlyBase
CG3216	–	None	Inferred membrane-associated guanyl cyclase receptor	FlyBase
For	Foraging	cGMP target	cGMP-dependent protein kinase	Osborne et al., 1997; MacPherson et al., 2004; Allen and Sokolowski, 2021; Kanoh et al., 2021
Pkg21D	Protein kinase, cGMP-dependent at 21D	cGMP target	cGMP-dependent protein kinase	Foster et al., 1996; Vermehren-Schmaedick et al., 2010
CG4839	–	cGMP target	cGMP-dependent protein kinase	Wu et al., 2020
CngA	Cyclic nucleotide-gated ion channel subunit A	cGMP target	cGMP-gated ion channel (plasma membrane)	Baumann et al., 1994
CngB	Cyclic nucleotide-gated ion channel subunit B	cGMP target	cGMP-gated ion channel (plasma membrane)	Finn et al., 1998
Cngl	Cyclic nucleotide-gated ion channel-like	cGMP target	cGMP-gated ion channel (plasma membrane)	Miyazu et al., 2000
CG42260	–	cGMP target	cGMP-gated ion channel (plasma membrane)	Vermehren-Schmaedick et al., 2010; Lee et al., 2020
Pde1c	Phosphodiesterase 1c	cGMP target	cGMP phosphodiesterase (cytosolic)	Day et al., 2005
Pde6	Phosphodiesterase 6	cGMP target	cGMP phosphodiesterase (cytosolic)	Day et al., 2005
Pde9	Phosphodiesterase 9	cGMP target	cGMP phosphodiesterase (cytosolic)	Day et al., 2005
Pde11	Phosphodiesterase 11	cGMP target	cGMP phosphodiesterase (cytosolic)	Day et al., 2005
Orco	Olfactory receptor co-receptor	?	Olfactory co-receptor (OR subsystem only)	Larsson et al., 2004; Benton et al., 2006
Or67d	Odorant receptor 67d	?	Pheromone receptor (at1 sensilla)	Kurtovic et al., 2007; Wang and Anderson, 2010
Ir8a	Ionotropic receptor 8a	?	Olfactory co-receptor (subset of IR subsystem only)	Benton et al., 2009; Abuin et al., 2011
Act5C	Actin 5C	None	Housekeeping, cytoskeleton component (pancellular)	Lü et al., 2018
Cam	Calmodulin	Constitutive NOS activation	Housekeeping, intermediate messenger (pancellular)	Regulski and Tully, 1995; Mukunda et al., 2016; Jain et al., 2021
Gapdh1	Glyceraldehyde 3 phosphate dehydrogenase 1	None	Housekeeping, glycolysis (pancellular)	Lü et al., 2018

**Figure 1:**
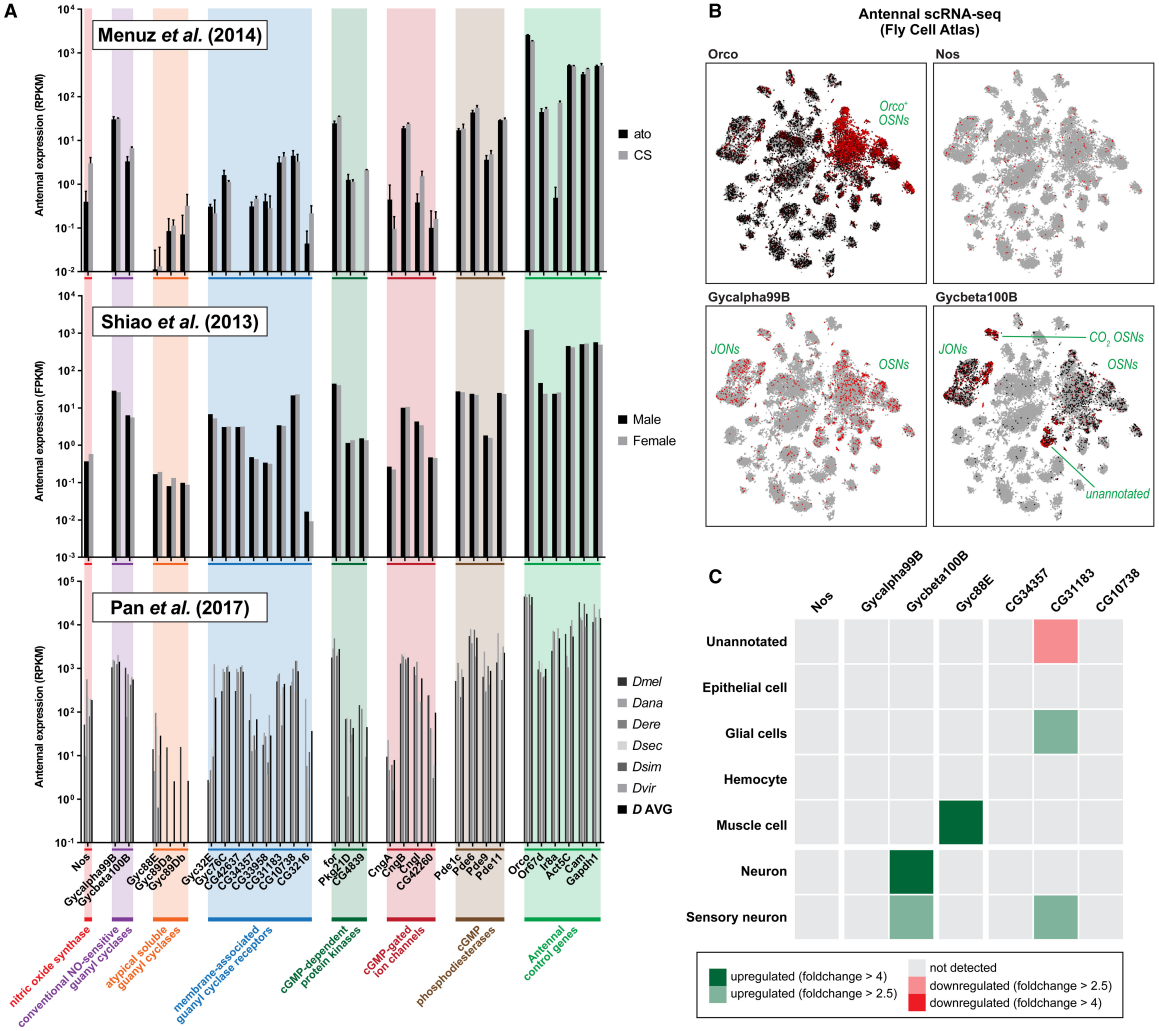
Antenna tissue-specific and antennal single-cell transcriptomics reveal neuronal expression of genes involved in the nitric oxide signaling pathway. **(A)** Expression of nitric oxide signaling pathway genes across three independent RNA-seq studies sampling antennal tissue only, showing abundant antennal expression of *Drosophila* nitric oxide synthase (Nos) and both conventional nitric oxide-sensitive guanyl cyclases (Gycα99B, Gycβ100B). Top panel: antennal single-end RNA sequencing of wildtype Canton-S (CS) and atonal (ato) mutant fly antennae, plotting normalized transcript expression in reads per kilobase million (RPKM) (Menuz et al., 2014). Both genotypes retain similar expression levels; notably, Nos is significantly but not entirely depleted in ato mutants which lack coeloconic but retain trichoid and basiconic sensilla. This suggests that the ionotropic receptor (IR) olfactory subsystem also expresses Nos. Middle panel: paired-end RNA sequencing of male and female flies, normalizing expression by fragments per kilobase million (FPKM) (Shiao et al., 2013). Both sexes display comparable expressions. Bottom panel: single-end RNA sequencing of six distinct species plotting normalized transcript expression in RPKM (Pan et al., 2017). A mean average across six species is also plotted (*D* AVG). Taken together, it is evident that a variety of fruit fly species exhibit comparable gene expression profiles within their antennae. **(B)** *t*-distributed stochastic neighbor embedding (tSNE) plots of antennal cells of the Fly Cell Atlas (Li et al., 2022) colored by gene expression (min-max) for four representative genes: Orco, Nos, Gycα99B, and Gycβ100B. Nitric oxide signaling genes show broad expression in Orco^+^ cells, which label Orco^+^ OSNs, the olfactory receptor (OR) subsystem. **(C)** Differential expression (DE) analysis of the above scRNA-seq dataset for some representative genes, by cell-type class compared to the complementary set of all other antennal cells. The nitric oxide-sensitive guanyl cyclase Gycβ100B is differentially expressed in antennal neurons; statistical testing and parametrization are outlined in Supplementary Figures 2, 3 and the Section 2. Colors indicate that statistically positive (green), negative (red), or insignificant (gray) differential expressions are detected at different fold-change thresholds (>4, dark; >2.5, light).

First, we consulted an RNA-seq study pooling 300 mixed-sex *D. melanogaster* flies, 5–12 days old post-eclosion, in both wildtype Canton-S flies and atonal (ato) mutants lacking coeloconic sensilla (Menuz et al., 2014). Dataset mining revealed abundant expression of genes involved in the NO signaling pathway, to amounts comparable with other genes of known antennal function (Figure 1A, top panel). In particular, Nos, Gycα99B, and Gycβ100B were more transcript-abundant than 32.6, 77.1, and 41.9% of all genes positively expressed in the wildtype antenna, respectively, and not among detected genes present only in trace amounts (Supplementary Figure 1). Although still present, the functional gene group with the relatively lowest expression abundance was the NO-insensitive atypical guanyl cyclases, which do not participate in the NO signaling cascade. Interestingly, ato mutants, which lack coeloconic but retain trichoid and basiconic sensilla, showed depleted but not absent antennal expression of Nos (~7-fold lower, *p* < 10^−8^, FDR < 10^−7^), suggesting that both ionotropic and olfactory receptor subsystems actively express nitric oxide synthase in adulthood.

Second, we consulted results from an RNA-seq study comparing gene expression between male and female sex. The study sampled 1,200 antennae pairs for male and female flies of age >1 day post-eclosion, in Canton-S flies separated by sex (Shiao et al., 2013). We found abundant expression of NO signaling pathway genes, corroborating the previous study, as well as no evident sexual dimorphism in transcript abundance (Figure 1A, middle panel).

Third, we consulted an RNA-seq study comparing antennal expression across drosophilid species boundaries (Pan et al., 2017), wherein expression abundance could be contrasted across closely and distantly related flies. Here, 300 mixed-sex antennae for six drosophilid species each (aged 7–10 days post-eclosion) were sampled. Transcriptome analysis yet again revealed positive expression of NO signaling pathway genes in all species' antennae, with little inter-species variation in gene expression (Figure 1A, bottom panel), suggesting that genes responsible for the production and reception of NO are ubiquitous across even distantly related *Drosophila spp*. such as *D. melanogaster* and *D. virilis*. Once more, between-gene expression profiles were found to corroborate well with other transcriptomes, indicating data robustness. For all studies consulted, we also plotted gene expression ordered by rank for all genes detected in antennae with labeled core genes of the NO signaling pathway (Supplementary Figure 1). As noted previously, all core NO signaling genes seem to be present in relative abundance and seem not to be negligible genes expressed in trace amounts.

Given the positive expression of NO signaling pathway genes in antennal tissue, we further looked at whether we could identify expression localization with the aid of antennal single-cell RNA-seq (scRNA-seq) data, a transcriptomic approach that retains cellular resolution in expression analysis. To this end, we consulted various antennal tissue datasets of the Fly Cell Atlas (Li et al., 2022). Although Nos transcripts were found to be scarce and unclustered, we found detectable neuron-biased expression of NO-sensitive guanyl cyclases in the antennal subset of cells, especially among Orco^+^ cells (Figure 1B), suggesting that Orco^+^ OSNs may harbor the receptors for any latent NO signals. Directly following, we expected the co-expression of at least some candidate genes involved in cGMP homeostasis or as cGMP effectors in the same cells as Orco. To test this hypothesis, we plotted the co-expression of Orco along with all candidate effector genes shortlisted previously. Here, we found substantial expression overlap between effector genes and Orco (Supplementary Figure 2). For stringency, we then also statistically tested whether these genes were more than 4-fold differentially upregulated in cells annotated as the “sensory neuron” or “neuron” class, relative to all other cells (i.e., non-neuronal cells) in the stringent antennal dataset produced by 10X microfluidic droplet-based single cell capture method. Here, Gycβ100B, Cngl, Pde1c, Pde6, and Pde9 were found significantly upregulated, while foraging (for) was the only downregulated candidate (Supplementary Figure 2B).

Finally, with hints that specific cells express machinery to facilitate and respond to NO signals, we asked which antennal cell type is enriched for core NO-signaling genes, with the expectation that these would be detectable above statistical threshold levels specifically in neurons following differential expression (DE) analyses. Indeed, we found transcript enrichment in neurons for the NO-sensitive guanyl cyclase subunit Gycβ100B, though were unable to find detectable enrichment of Nos and Gycα99B (Figure 1C). As a control, a sample of functionally related genes such as membrane-associated and atypical soluble guanyl cyclases not sensitive to NO did not show similar neuronal enrichment (Figure 1C). As an additional check, we expanded the analysis to consider finer-grained subcategorizations in the dataset by cell subtype and scRNA-seq tissue isolation method. Here, we found Gycβ100B enriched in expression in a patchy manner across olfactory sensory neuron subtypes and also overrepresented in Johnston organ mechanosensory neurons (Supplementary Figure 3). By looking into the antennal dataset generated by the plate-based SMART-seq2, which yields increased gene detection as a result of higher sequencing depth, and thus facilitating cell-specific detection of lowly expressed genes (Li et al., 2022), we also found a first instance of Nos and Gycα99B enrichment among the Ir58a^+^ Orco^−^ OSN annotated cell group (Supplementary Figure 3).

In sum, there are several lines of molecular evidence showing that machinery for producing, receiving, and terminating NO signals is present in *Drosophila* antennae, as well as some tentative hints that necessary elements such as core pathway genes such as nitric oxide synthase and the NO-sensitive soluble guanyl cyclases are enriched within olfactory neurons, suggesting that the pathway may be involved in this sensory organ.

### 3.2. Calcium imaging in an open antenna stimulated with odor proxy VUAA1 as a measure of olfactory response

**Figure 2:**
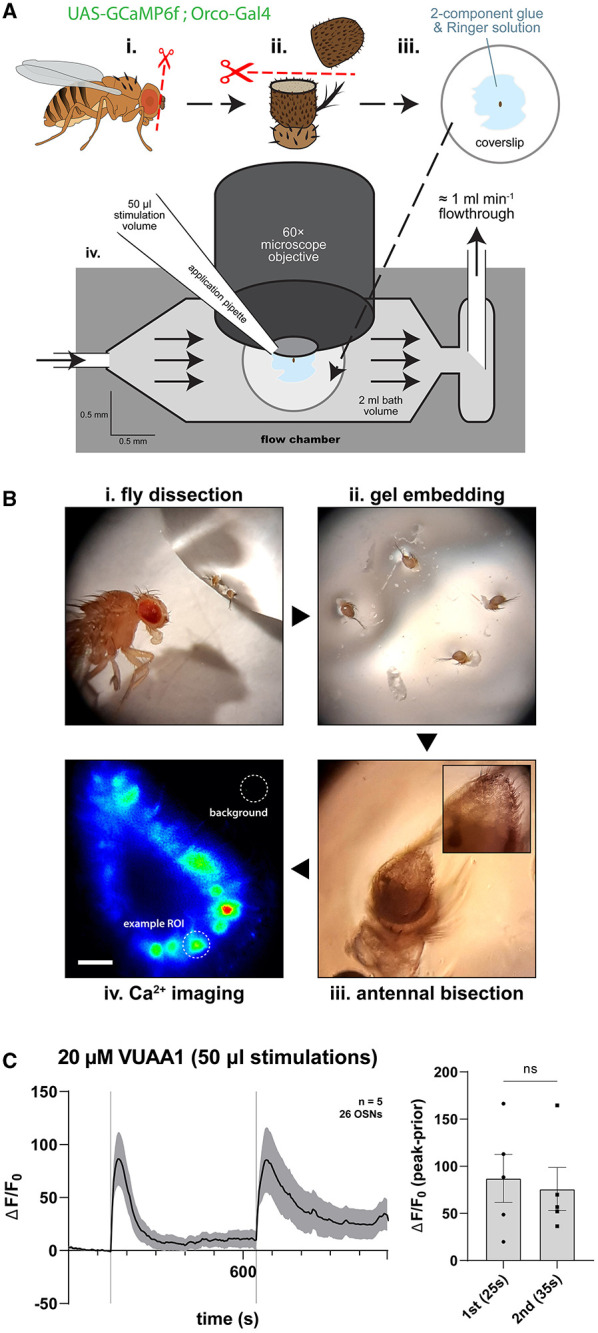
Experimental workflow of *ex vivo* Ca^2+^ imaging of *Drosophila* olfactory sensory neurons using a continuous flow chamber. **(A)** Antennae of *D. melanogaster* flies expressing Ca^2+^ sensor GCaMP6f in Orco^+^ olfactory sensory neurons are excised and deposited into *Drosophila* Ringer solution (i), then oriented and held in place within a two-component hydrogel (ii) before bisection using micro-scissors (iii). This reveals the funiculus interior for live cell imaging with fluorescence microscopy within a flow chamber delivering continuous flowthrough of Ringer solution during pulsed application of chemicals onto the exposed antennal cells via pipetting (iv). **(B)** Exemplar micrographs of the steps illustrated above. The final micrograph is heat-colored by fluorescence intensity and demonstrates a background selection and a typical OSN region of interest (ROI) selection for which the fluorescence signal is traced. Scale bar: 10 μm. **(C)** Average time course plot showing Ca^2+^ dynamics during a standard protocol featuring two OSN stimulations with the Orco agonist VUAA1 (vertical lines). The bar plot indicates the mean average total response amplitude for each peak response ± SEM with peak response latencies reported in parentheses.

To address whether the function of olfactory sensory neurons may be modulated by nitric oxide or the products of its signaling cascade, we performed liquid phase odor stimulation and fluorescence imaging of *Drosophila* antennae as done previously (Mukunda et al., 2014, 2016; Miazzi et al., 2016; Halty-deLeon et al., 2018, 2021; Jain et al., 2021). We devised a flow chamber approach on *ex vivo* antennal preparations of *D. melanogaster* antennae, wherein dissected and subsequently bisected antennal preparations maintained in a physiological Ringer solution allowed optical and pharmacological access, thus allowing for quantifying time-separated olfactory responses to extraneous applications of the odor proxy VUAA1 (Figures 2A, B). VUAA1 is a synthetic, non-competitive, and allosteric agonist of Orco (Jones et al., 2011), a functional component of the OR complex that abundantly localizes to the dendrites of OSNs. Upon application, OR complexes are activated by VUAA1, which opens and passes cations including Ca^2+^. OR activation is thus accompanied by an increase in [Ca^2+^]_i_ (Wicher et al., 2008; Mukunda et al., 2014). In the experimental design, we utilized the OSN-restricted expression of the Ca^2+^ sensor GCaMP6f (Chen et al., 2013) using the binary GAL4/UAS expression system to monitor OR activation on a cell-by-cell basis.

As a standard control for subsequent experiments, we determined the concentration and application volumes of VUAA1 to assure that stimulations separated in time induced identical responses, occurring from similar baselines of neuronal resting state, as well as produced non-saturated responses. Here, we found that stimulation-induced neuronal responses to the application of 50 μl 20 μM VUAA1 were appropriate, where both responses exhibited comparable response amplitudes (Figure 2C). We note that the tail of the second response may not return to baseline levels comparable to those following the first response, a feature which may be attributed to Ca^2+^ store filling and subsequent release as previously described for mitochondria (Lucke et al., 2020; Wiesel et al., 2022). The presence of the intermediate solvent for VUAA1, DMSO, was also found to elicit no response previously (Prelic et al., 2022). This standard is used as the basis for subsequent experiments comparing response magnitudes in the presence or absence of pharmacological modulations.

### 3.3. Inhibition and activation of the NO-cGMP pathway has no effect on OSN responses

**Figure 3:**
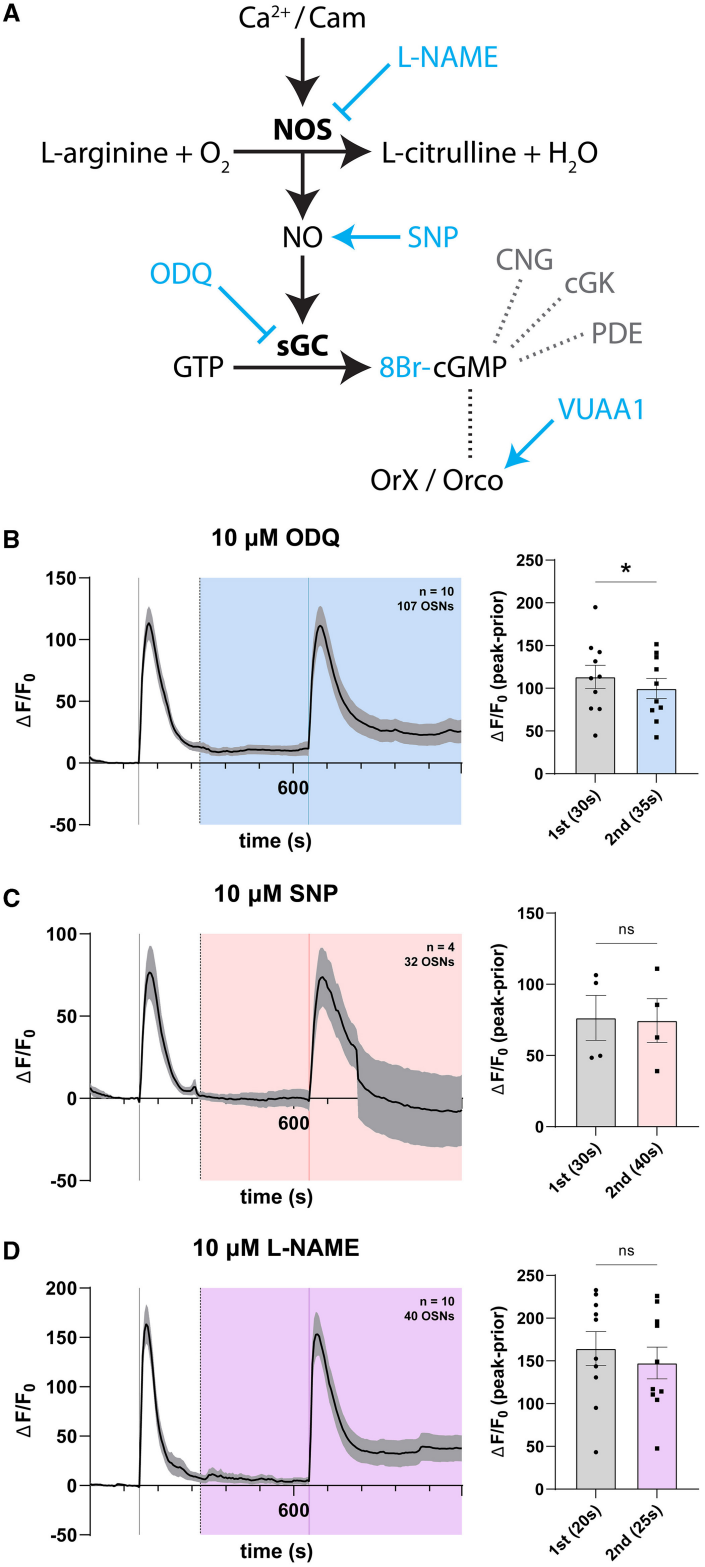
Modulation of the nitric oxide signaling pathway has no effect on OSN response. **(A)** Schema of the nitric oxide signaling pathway. Activation of nitric oxide synthase (NOS) induces nitric oxide (NO) production. In turn, NO activates soluble guanyl cyclase-dependent catalysis of GTP into cGMP, which is hypothesized to induce non-selective channel opening of Orco. Various agents (blue) can be used to modulate the signaling pathway. NO signaling leads to increased production of the cyclic nucleotide cGMP. cGMP in turn acts on cyclic nucleotide-gated ion channels (CNG), regulates cGMP-dependent protein kinases (cGK), and gets degraded by specific phosphodiesterases (PDE). **(B)** Time course of OSN responses to VUAA1 in the absence (first response) and presence (second response) of 10 μM ODQ, a highly selective and irreversible inhibitor of soluble guanyl cyclase. The bar plot indicates the mean total response amplitude for each response ± SEM. **(C)** Time course of OSN responses in the absence (first response) and presence (second response) of 10 μM sodium nitroprusside (SNP), a potent nitric oxide donor functioning as an activator of soluble guanyl cyclase. The bar plot indicates the mean total response amplitude for each response ± SEM. **(D)** Time course of OSN responses in the absence (first response) and presence (second response) of 10 μM L-NAME, a nitric oxide synthase inhibitor. The bar plot indicates the mean total response amplitude for each response ± SEM. Peak response latencies are reported in parentheses in all bar plots.

Given hints of expression of core NO signaling genes in the antenna, we asked whether NO signaling might play a specific role in modulating olfactory responses in the sensory periphery of *Drosophila*, as a mode of intra- or intercellular signaling with effects observable in sensory neuron responses. To address this, we devised a set of paired-stimulation experiments wherein control responses to the OR agonist VUAA1 were recorded first, and then compared in the same cells with responses occurring in the presence (under continuous perfusion) of disrupting or stimulating agents of key enzymes of the signaling pathway. The interstimulation interval was chosen to allow for complete relaxation of the first response as well as complete perfusion of modulators. We selected to interfere with the NO-cGMP pathway at several stages (Figure 3A). Activation or inhibition of key components of the NO-cGMP cascade was achieved with the use of pharmacological compounds previously validated in insect and *Drosophila* systems, which have been shown to potentiate NO signaling and exhibit bioactivity at concentrations of 10 μM in cockroach neurons (Wicher et al., 2004), *Drosophila* S2 cell lines and incubated *Drosophila* central nervous systems (Gibbs and Truman, 1998; Dijkers and O'Farrell, 2009), and in neuromuscular junctions (Wildemann and Bicker, 1999) and Malpighian tubules of *Drosophila* (Dow et al., 1994; Broderick, 2002).

First, we compared olfactory responses to VUAA1 stimulations within antennae in the presence and absence of ODQ, an irreversible inhibitor of soluble guanyl cyclase (Garthwaite et al., 1995; Schrammel et al., 1996; Nighorn et al., 1999; Gibbs, 2001; Langlais et al., 2004; Morton, 2004; Morton et al., 2005). We found a significant though marginal decrease in peak response in the presence of 10 μM ODQ (Figure 3B). Following this observation, we similarly recorded responses in the presence and absence of 10 μM sodium nitroprusside (SNP), a ferrous iron complexed with NO, which acts as a non-selective NO donor and activator of sGC (Katsuki et al., 1977; Nighorn et al., 1999; Langlais et al., 2004). Here, we found no change in OSN response (Figure 3C). Finally, we recorded responses in the presence of 10 μM L-NAME, a nitric oxide synthase inhibitor (Rees et al., 1990; Furfine et al., 1993; Gibbs, 2001), and also found no detectable effect on OSN response (Figure 3D). The time to reach maximum response peak was also not significantly different for stimulations in the presence of any of these NO signaling modulators, nor different between first and second stimulations (Supplementary Figure 4).

### 3.4. The cyclic nucleotide cGMP has no effect on the responses of OSNs in their native antennal environment

**Figure 4:**
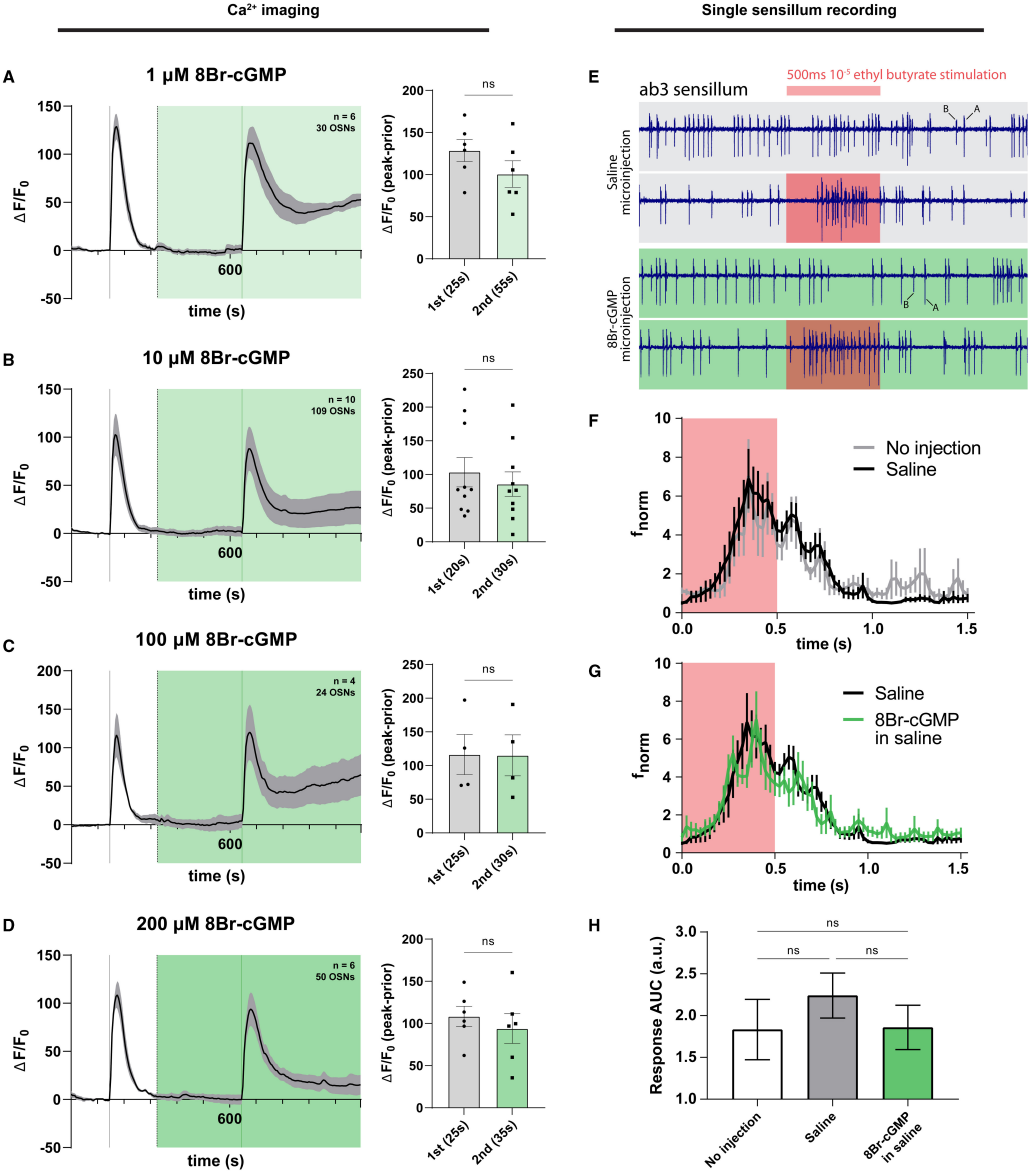
cGMP does not affect OSN responses in a long exposure regime and when microinjected into sensilla. Olfactory responses were surveyed using Ca^2+^ imaging of OSNs (left column) and single sensillum recording of the ab3A neuron (right column). **(A)** Time course of OSN responses to VUAA1 in the absence (first response) and presence (second response) of 1 μM 8Br-cGMP, a cell-permeable, hydrolysis-resistant analog of cGMP. The bar plot indicates the mean total response amplitude for each response ± SEM. **(B)** Time course of OSN responses in the absence (first response) and presence (second response) of 10 μM 8Br-cGMP, a cell-permeable, hydrolysis-resistant analog of cGMP. The bar plot indicates the mean total response amplitude for each response ± SEM. **(C)** Time course of OSN responses in the absence (first response) and presence (second response) of 100 μM 8Br-cGMP, a cell-permeable, hydrolysis-resistant analog of cGMP. The bar plot indicates the mean total response amplitude for each response ± SEM. **(D)** Time course of OSN responses in the absence (first response) and presence (second response) of 200 μM 8Br-cGMP, a cell-permeable, hydrolysis-resistant analog of cGMP. The bar plot indicates the mean total response amplitude for each response ± SEM. **(E)** Representative single sensillum recording (SSR) traces of ab3 sensilla in a saline vehicle-microinjected sensillum (top) and 8Br-cGMP microinjected sensillum (bottom). Red intervals denote a 500 ms duration of 10^−5^ ethyl butyrate stimulation. A and B labels indicate neuron spike activity of spike magnitude-distinguishable large A and small B neurons contained within ab3 sensilla. **(F)** Normalized frequency response (f_norm_) of ab3A neuron to 500 ms stimulation using 10^−5^ ethyl butyrate, measured via single sensillum recording during microinjection of saline (“Saline”) or without any microinjection (“No injection”) into the corresponding sensillum lymph. Frequencies correspond to neuronal responses of the ab3A neuron binned using 25 ms intervals. **(G)** Normalized frequency response (f_norm_) of ab3A neuron to 500 ms stimulation using 10^−5^ ethyl butyrate, measured via single sensillum recording during microinjection of saline vs. 8Br-cGMP (in saline) into the sensillum lymph space. Frequencies correspond to ab3A neuronal responses binned using 25 ms intervals. **(H)** The area under the curve quantification of OSN responses (response AUC) is shown in preceding panels E and F. For response frequency time plots, the area under the curve was obtained as an approximation of the total number of spikes within the immediate response window (defined as 500 ms during and 1,000 ms following stimulation with 10^−5^ ethyl butyrate). To compare data, an ordinary one-way ANOVA with Dunnett's multiple comparison test was performed. Bars indicate mean AUC ± SEM. Peak response latencies are reported in parentheses in all bar plots.

Given that we could not reliably detect a contribution of the NO signaling pathway to modulating the magnitude of olfactory responses in antennal OSNs, we set out to more rigorously test whether the cascade's terminal effector, cGMP, would have any effect on olfactory responses. Plated HEK293 cells heterologously expressing the *Drosophila* Orco protein have been previously shown to respond to cAMP and cGMP (Wicher et al., 2008). As cAMP was seen to potentiate OSN activity (Getahun et al., 2013), one could expect that cGMP would also have potentiating effects on olfactory responses. To test this in the native context of the antenna, we exposed *ex vivo* antennal preparations to differing concentrations of 8-bromo-cGMP (8Br-cGMP), a cell-permeable analog of endogenous cGMP that is resistant to degradation via hydrolysis by cGMP phosphodiesterases (Rapoport et al., 1982; Gibbs, 2001). No significant effect of cGMP on olfactory responses was observed across three physiologically relevant orders of concentration magnitude, in neither experiment involving olfactory stimulations featuring long-term exposures of the antenna to 1 μM 8Br-cGMP (Figure 4A), 10 μM 8Br-cGMP (Figure 4B), 100 μM 8Br-cGMP (Figure 4C), and 200 μM 8Br-cGMP (Figure 4D). Likewise, the time to reach the maximum response peak was not significantly different for stimulations in the presence or absence of any concentration of 8Br-cGMP, nor different between first and second stimulations (Supplementary Figure 4).

Given the lack of effect across a wide range of concentrations in a long-term exposure context, we reasoned that the effect might be restricted temporally to shorter time scales unobservable using fluorescence Ca^2+^ imaging, which has a low temporal resolution that is unable to discern short-lived or immediate effects. To observe potential differences in acute olfactory responses with a higher temporal resolution, we opted to use the electrophysiological technique of single sensillum recording (SSR) during piezo-controlled microinjection of 8Br-cGMP into the sensillum lymph, as performed previously (Olsson et al., 2011; Getahun et al., 2013). This would additionally have the added benefit of assuring the delivery of the experimental analog of cGMP to the site of odor transduction, the sensillum lymph itself, as well as stand as an experiment performed *in vivo* without requiring the excision of tissue for fluorescence imaging.

We selected the large A neuron of the well-studied large basiconic ab3 sensillum for electrophysiological testing, which harbors the Or22a/Or22b olfactory receptors responsive to ethyl butyrate, among other odorants, given the broad tuning of these receptors (Dobritsa et al., 2003; Shaw et al., 2019). By using ethyl butyrate stimulations, we hypothesized that olfactory responses would be different under 8Br-cGMP microinjection conditions in a manner specific to the A neuron. First, to rule out the confounding effects of microinjection itself, we determined that our microinjection paradigm would leave olfactory responses intact and unaffected by the microinjection protocol. We did this for three measures: peak response frequency, time-to-peak response, as well as total spike count as determined by measuring area-under-curve (AUC) for the response frequency within the initial 1,500 ms following stimulation onset. Here, we considered the A neuron only. We found no difference in response strength (as measured by area under the frequency curve) across treatments, between uninjected and saline-microinjected conditions (Figure 4E; Supplementary Figure 5A).

Next, we compared microinjections of a saline control with 8Br-cGMP in saline and surprisingly found no observable effect on response dynamics and kinetics (Figure 4F). Likewise, no difference in response latency was observed (Supplementary Figure 5B). This was unexpected given that the sister cyclic nucleotide cAMP has been shown to increase olfactory responses of ab3A in the same setup (Getahun et al., 2013) and has been demonstrated in several studies to potentiate responses through its action on the olfactory receptor complex (e.g., Wicher et al., 2008; Dolzer et al., 2021).

Finally, to quantify the total electrophysiological response to ethyl butyrate stimulation, we calculated the total spike count as determined by measuring AUC for the response frequency within the initial 1,500 ms following stimulation onset. Using this quantification, we also found no differences between the presence or absence of 8Br-cGMP and the no-microinjection control (Figures 4G, H), thus indicating that cGMP seems not to play a role in the size or timing of acute responses to an ecologically relevant odor such as ethyl butyrate. Interestingly, this is distinct from identically carried out experiment involving microinjections of 8Br-cAMP, which has previously been reported to increase olfactory responses, at least in ab3A neurons in response to ethyl butyrate (Getahun et al., 2013) (Supplementary Figure 5). The results are, however, in agreement with the preceding calcium imaging observations, where no effect on response is discernable.

Based on the synthesis of observations made using calcium imaging and sensillum microinjections during electrophysiological recording, we conclude that cGMP has no potentiating effect on olfactory responses when exposed to cGMP over long periods. The result stands in contrast with cAMP, which has been shown previously to increase immediate response amplitude to odorants in single sensillum recordings and to contribute to olfactory response sensitivity (Getahun et al., 2013).

### 3.5. *Ex vivo* modulation of the cAMP pathway shows no effect on olfactory responses

**Figure 5:**
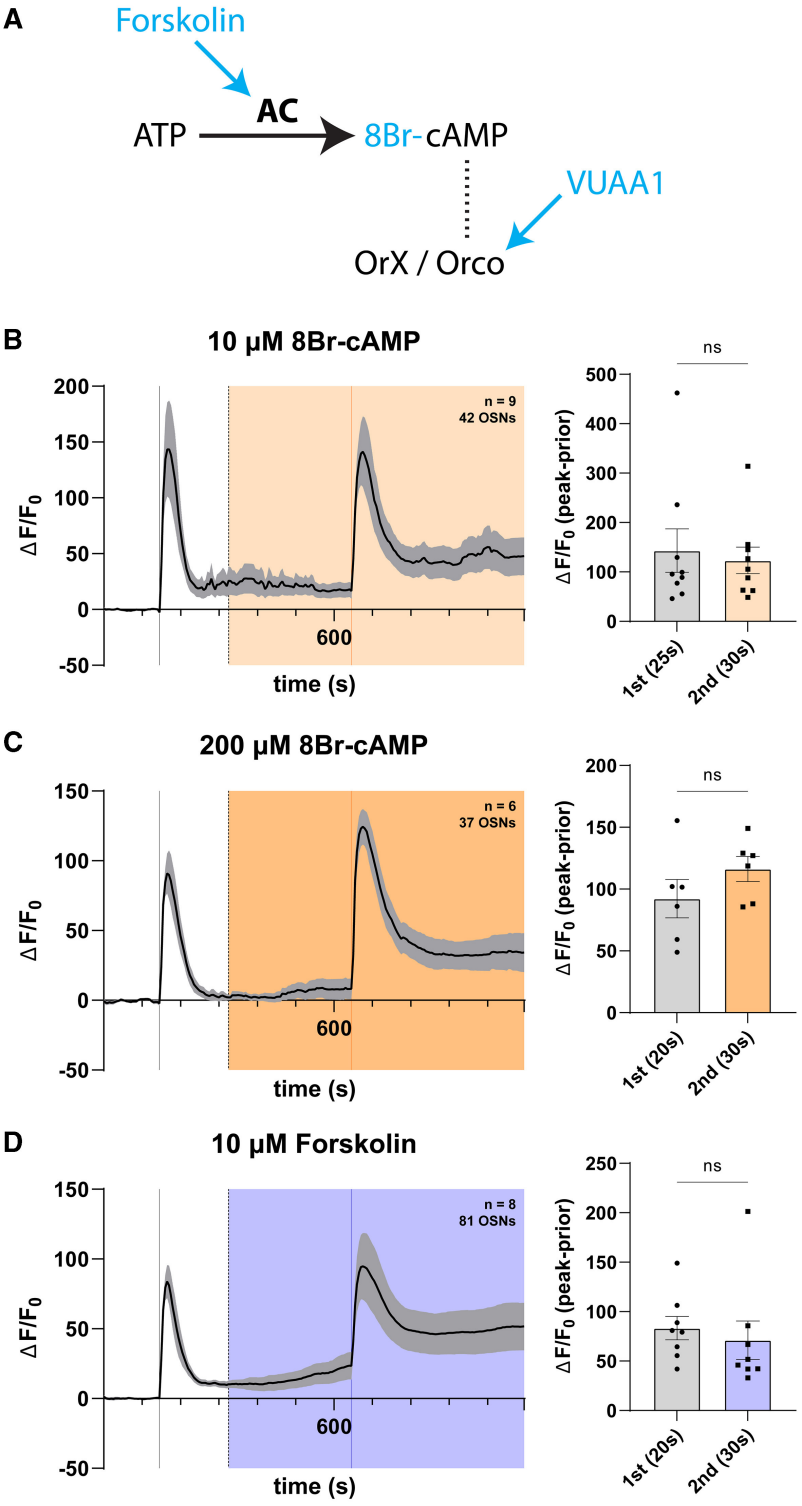
cAMP does not modulate OSN responses in a long exposure regime. **(A)** Schema of the cAMP pathway. Adenyl cyclase (AC) catalyzes the conversion of ATP into cAMP, which is hypothesized to induce Orco channel opening through direct binding. Various pharmacons (blue) can be used to modulate this pathway. **(B)** Time course of OSN responses to VUAA1 in the absence (first response) and presence (second response) of 10 μM 8Br-cAMP, a cell-permeable, hydrolysis-resistant analog of cAMP. The bar plot indicates the mean total response amplitude for each response ± SEM. **(C)** Time course of OSN responses in the absence (first response) and presence (second response) of 200 μM 8Br-cAMP, a cell-permeable, hydrolysis-resistant analog of cAMP. The bar plot indicates the mean total response amplitude for each response ± SEM. **(D)** Time course of OSN responses in the absence (first response) and presence (second response) of 10 μM forskolin, a potent adenyl cyclase agonist (activator). The bar plot indicates the mean total response amplitude for each response ± SEM. Peak response latencies are reported in parentheses in all bar plots.

In the context of heterologous expression of olfactory receptors, cyclic nucleotides activate Orco (Wicher et al., 2008; Sargsyan et al., 2011), as well as the olfactory receptor complex in some contexts (Deng et al., 2011; Olsson et al., 2011; Getahun et al., 2013; Miazzi et al., 2016). Given that we found no olfactory response effects of cGMP applications to OSNs in their native environment, we next asked whether cAMP specifically could exert increased responses in a long-term exposure paradigm as used previously, given previous reports that microinjections of cAMP or cAMP-producing agents increase responses as assayed by single sensillum recording (Supplementary Figure 5) (Getahun et al., 2013). For this purpose, we selected several approaches to modulate cAMP availability in OSNs by exploiting the cAMP pathway, which purportedly acts on Orco as one of its terminal effectors (Figure 5A). We devised two previously explored modes. First, we directly applied 8-bromo-cAMP (8Br-cAMP) into the bath chamber, an analog of endogenous cAMP previously shown to play a role in the sensitization of olfactory responses following repeated odorant stimulations (Getahun et al., 2013; Mukunda et al., 2016). This effect of cAMP is dependent on the presence of Orco, which is required to be sufficiently phosphorylated by PKC to allow for its activation by cAMP, as well as to mount an observable sensitization to repeated stimulations (Sargsyan et al., 2011). Second, we applied forskolin, a cell-permeable activator of transmembrane adenyl cyclases, to the open antenna. Adenyl cyclase is a specific enzyme catalyzing conversion of ATP to cAMP (Alasbahi and Melzig, 2012; Miazzi et al., 2016). The action of forskolin within the cAMP pathway is shown in Figure 5A.

As with preceding Ca^2+^ imaging experiments involving 8Br-cGMP, we first applied 10 μM 8Br-cAMP to the solution bathing open antennal preparations and found no effect on response magnitudes (Figure 5B). We subsequently attempted stimulations in the presence of a much higher concentration of 200 μM 8Br-cAMP and similarly found no difference in response magnitude (Figure 5C). We reasoned that long-term application of 8Br-cAMP might saturate any dynamic cAMP-dependent action it may have on olfactory responses. As an alternative, we attempted the same by surveying VUAA1 responses in the presence of 10 μM forskolin, hypothesizing that cAMP generated in an intracellular fashion by existing machinery may allow for potentiated responses. Here, we also found no difference in response magnitude between control stimulation and in the presence of forskolin, though found observable increases in baseline activation of OSNs (Figure 5D), a response to forskolin reported previously (Miazzi et al., 2016), indicating an ongoing biological response to induced and accelerated production of intracellular cAMP. Likewise, the time to reach the maximum response peak was not significantly different for stimulations in the presence or absence of any concentration of 8Br-cAMP, nor different between the first and second stimulations (Supplementary Figure 4).

In sum, we were unable to detect any olfactory response-modulating effect of cAMP. This parallels the lack of effect observed with previous experiments involving applications of cGMP, another cyclic nucleotide implicated in olfactory receptor complex regulation.

### 3.6. Modulation of the cAMP but not cGMP pathway shortly before OSN stimulation elicits response effects

**Figure 6:**
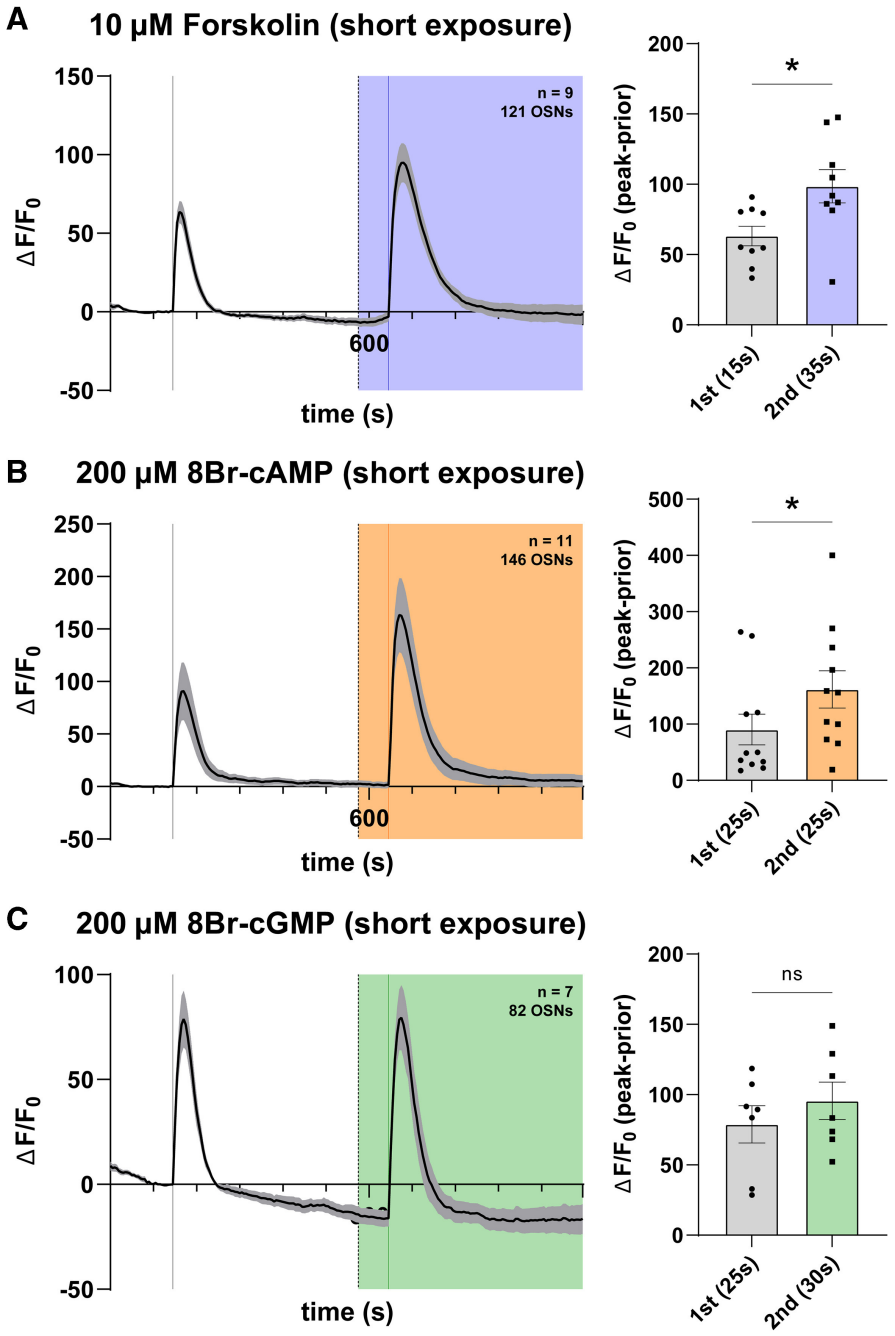
cAMP but not cGMP affects olfactory response in a short-term regime. **(A)** Time course of OSN responses to VUAA1 in the absence (first response) and presence (second response) of 10 μM Forskolin applied shortly before (70 s) testing of an olfactory response. Relative response magnitude is increased in the presence of forskolin applied shortly before stimulation (*p* = 0.01). The bar plot indicates the mean total response amplitude for each response ± SEM. **(B)** Time course of OSN responses in the absence (first response) and presence (second response) of 200 μM 8Br-cAMP applied shortly before (70 s) testing of an olfactory response. Relative response magnitude is increased in the presence of 8Br-cAMP applied shortly before stimulation (*p* = 0.03). The bar plot indicates the mean total response amplitude for each response ± SEM. **(C)** Time course of OSN responses in the absence (first response) and presence (second response) of 200 μM 8Br-cGMP applied shortly before (70 s) testing of an olfactory response. Relative response magnitude is unchanged in the presence of 8Br-cAMP applied shortly before stimulation (*p* = 0.81). The bar plot indicates the mean total response amplitude for each response ± SEM. Peak response latencies are reported in parentheses in all bar plots.

Previous evidence suggests that sensitization in *Drosophila* OSN responses is restricted temporally, namely that repeat presentation of an odor at subthreshold concentration with a 3-min interstimulus interval fails to elicit sensitization, as surveyed by single sensillum recording (Getahun et al., 2013). However, when odors are presented with shorter interstimulus intervals, sensitization occurs, and the degree of sensitization is proportional to cAMP levels (Getahun et al., 2013). This may be interpreted as a relevant time frame for cyclic nucleotide action on OSNs *in vivo*. To address the potential confoundment, given that all previous Ca^2+^ imaging experiments feature cyclic nucleotide or modulator exposures much longer than 5 min (i.e., 320 s), we repeated our experiments wherein cyclic nucleotide analogs were presented more briefly before OSN stimulation by VUAA1. Here, we selected to apply cAMP pathway modulators forskolin and 8Br-cAMP at identical concentrations as previously, but this time 70 s before stimulation in a “short exposure” paradigm. We found increased response magnitude in the presence of both 10 μM forskolin (Figure 6A) and 200 μM 8Br-cAMP (Figure 6B), in line with previous findings. To determine whether the same applied to the cGMP pathway, we separately applied 8Br-cGMP 70 s before stimulation but found no hint of a response effect under any concentration, including 200 μM 8Br-cGMP (Figure 6C). We, therefore, find a quantitative difference in response-modulating action between cAMP and cGMP in short-term exposure experiments, but once again observe no detectable action of cGMP on olfactory responses.

## 4. Discussion

Using pharmacological manipulation of the NO-cGMP pathway, we found no change in olfactory responses in *Drosophila* antennal preparations. This conclusion is based on two lines of evidence. First, by inhibiting or activating NO signaling machinery, we found no reliable difference in olfactory responses among Orco^+^ OSNs in a repeated stimulation experiment design utilizing *ex vivo* antennal Ca^2+^ imaging. Although first observing a marginal but significantly increased response effect during an sGC inhibition condition, we were unable to reproduce this trend with the use of other agents attenuating intracellular cGMP levels. Similarly, we covered a wide range of physiologically relevant cGMP concentrations (Ignarro, 1974; Rashatwar et al., 1987; Buchan and Martin, 1991; Sekhar et al., 1992; Choi and Farley, 1998; Morton et al., 2005), as well as different time frames related to perfusion of modulators onto antennal tissue, and found no neuronal response effects with regard to amplitude or latency. Most crucially, no effect was observed during activation and inhibition of NOS, which has shown effects at comparable concentrations elsewhere in *Drosophila* tissues and systems (Dow et al., 1994; Gibbs and Truman, 1998; Wildemann and Bicker, 1999; Gibbs, 2001; Broderick, 2002; Dijkers and O'Farrell, 2009). Second, by microinjecting the NO signaling pathway's terminal effector cGMP into pre-defined sensilla, we similarly found no detectable response effects, an observation which starkly contrasts with that of cAMP microinjections at identical concentrations, wherein increased OSN responses are apparent (Getahun et al., 2013). Taken together, we conclude that NO signaling plays no overt role in potentiating responses to odor cues.

Although our findings are well-supported by various observations, we note that a lack of observed response effect may be confounded if NO signaling was heavily restricted to a small subpopulation of OSNs. This point is presently motivated in three ways. First, the GAL4/UAS system used herein targets GCaMP expression to Orco^+^ cells, which do not make up the entirety of OSNs, but rather only constitute the majority that excludes the ionotropic receptor (IR) subsystem, where Orco does not serve constitutively as OR complex co-receptor. Second, stainings and localization of NOS and NO receptors in OSNs have been reported to be expressed in patchy or restricted manners in several cases, such as in cuttlefish, where NOS immunoreactive staining is confined to interspersed punctata in the olfactory organs, suggesting that NO is utilized for neurotransmission in only a subset of OSNs (Scaros et al., 2018). Likewise, specific sGCs such as Gucy1b2 are found in specific zones of the olfactory mucosa in rodents and zebrafish (Ruiz Tejada Segura et al., 2022), where they have been suggested to participate in NO signaling in a patchy manner (Saraiva et al., 2015). Third, our present bioinformatic analysis of both antennal and single cell-resolving RNA-seq datasets of *Drosophila* antennal tissue indicates that gene transcripts of NO signaling pathway machinery are not uniformly distributed across antennal cells nor OSNs. As mentioned previously, the IR subsystem is NOS enriched, as is evidenced by Nos depletion in ato mutants which lack coeloconic sensilla characteristic of the IR subsystem (Menuz et al., 2014); in effect, these mutants are OR subsystem enriched and IR subsystem deficient, which implies that NOS may be likelier to function in the Orco^−^ IR subsystem. However, whether this holds is unclear, as more recent comparative transcriptomic studies comparing antennae from ato and amos mutants reported no detectable differential expression in NOS (Nos) and sGC (Gycα99B, Gycβ100B) genes between ato mutants (which lack the IR subsystem) and amos mutants (which lack the OR subsystem) above a >4-fold expression threshold (Scalzotto et al., 2022). Likewise, in a similar study specifically comparing antennae of wildtype flies and amos mutants, Nos is also found differentially depleted in amos mutant antennae lacking basiconic and trichoid sensilla of the OR subsystem (Mohapatra and Menuz, 2019). Taken together, this suggests that NOS is expressed within both olfactory subsystems. In our study, when considering single antennal cell transcriptomes, we noted a detectable difference in NOS expression only in the annotated cell group of Ir58a^+^ Orco^−^ OSNs. We, therefore, remark that this study does not address the possibility of NO signaling having effects on olfactory responses within the IR subsystem, as it is not probed by a survey of the Orco^+^ subset of OSNs. As such, the current experimental paradigm cannot conclusively test putative modes of regulation within the IR subsystem. A potential resolution to conflicting molecular data is the employment of protein staining techniques: for instance, NOS can be histochemically labeled via NADPH diaphorase staining (Gonzalez-Zulueta et al., 1999), a successful staining performed previously in *Drosophila* (e.g., Müller, 1994). However, it is not a foolproof technique for detecting NOS, as NOS-deficient animals can show positive labeling as the technique is reliant on a diaphorase reaction revealing NADPH oxidation, which is often co-incident with NOS, though not always attributable to NOS activity (Gonzalez-Zulueta et al., 1999). Alternatively, the use of peptide fragment-derived anti-*Drosophila* NOS antibodies have been used with success in *Drosophila* larvae and development studies previously (Yakubovich et al., 2010; Lacin et al., 2014), as well as in the *Drosophila* central brain (Kuntz et al., 2017). sGC immunolabeling has been attempted in chemosensory sensilla in the adult locust, where immunoreactivity was found in somata of chemoreceptors within basiconic sensilla on the distal femur (Ott et al., 2000), a result yet to be replicated in *Drosophila*, though which is beyond the scope of our antennal study. Finally, the manipulation of NOS activity may also be achieved via implementing routine binary expression systems in *Drosophila*, such as Gal4/UAS, wherein Nos-promoter Gal4 elements can be used to express reporters or activity-controlling genes such as optogenetic tools in a manner restricted to cells with NOS activity.

Another hallmark difficulty in the field is following concentrations of signaling molecules such as cyclic nucleotides during the signal transduction event, or in tracing Ca^2+^ and cyclic nucleotide levels simultaneously. The establishment of a variety of fluorescent indicators is ongoing, though few attempts in the field of olfactory transduction have been made in this direction. For instance, studies employing FRET-based fluorescent cAMP sensor Epac1-camps have permitted cAMP level quantification during olfactory responses in *Drosophila* antennae (Miazzi et al., 2016). To overcome this presently, we tested olfactory responses across a wide concentration range of cyclic nucleotides. In a similar vein, we suggest future studies adopt available genetically encoded cGMP sensors for *Drosophila*. The recent development of novel intracellular cGMP sensors (Matsuda et al., 2017; Calamera et al., 2019) may now constitute avenues for co-imaging of both neuronal activation and cGMP levels simultaneously, though this is yet to be validated for *Drosophila* and would require the generation of new transgenic lines.

Although we found hints of antennal expression of NO signaling pathway genes but no evidence of the functional effect of modulating NO signals on odor responses, we cannot exclude the occurrence of antennal NO signaling based on functional cation imaging and electrophysiological recordings. If the expression of NO signaling genes is actively maintained into insect adulthood, and NO signaling indeed occurs, three explanations of our observations are possible.

First, NO signaling may not play a role in the antennal locale or at the chemoreceptor level, but rather expressed components of the signaling pathway such as NOS may be trafficked in an anterograde fashion from the antenna through OSN axonal projections to function distally in synaptic termini at the antennal lobe, where NO signaling has been shown to occur at least in *Manduca* (Collmann et al., 2004), honeybees (Müller and Hildebrandt, 1995), *Drosophila* (Müller and Buchner, 1993), and *Schistocerca* locusts (Müller and Bicker, 1994), though only in the latter case, the NADPH-diaphorase NOS-specific staining derives specifically from antennal lobe interneurons rather than OSNs. For instance, non-overlapping immunocytochemical labeling for sGC and NOS in *Manduca* antennal lobes has been reported, where afferent OSN axons were found NOS-immunoreactive (Collmann et al., 2004). Alternatively, NO signaling may function in cells not immediately adjacent to OSN dendrites in *Drosophila*. Examples include perineural glia, whose projections are not restricted to the funiculus (Sen et al., 2005; Calvin-Cejudo et al., 2023), and cells found within the preceding (first and second) antennal segments, which are co-sampled along with OSNs of the third antennal segment in RNA-seq studies but which play no role in olfactory sensing. Regarding the latter, the second segment harbors the Johnston's organ, a mechanosensor responsible for *Drosophila* hearing, gravity-sensing, and proprioception (Kamikouchi et al., 2009; Boekhoff-Falk and Eberl, 2014). This explanation is motivated by two independent observations of sGC-positive antennal mechanosensory axons in the vicinity of *Manduca* and *Schistocerca* antennal lobes (Elphick and Jones, 1998; Collmann et al., 2004), which implicate the mechanosensory system as a site for NO signaling. Finally, NO signaling may not be related to olfactory but rather an immune function, as is the case in *Drosophila*, where it contributes to innate immune responses to microbes following infection (Foley and O'Farrell, 2003; Lemaitre and Hoffmann, 2007; Carton et al., 2009; Inamdar and Bennett, 2014). Inducible synthesis of NO has long been shown to occur in several mosquito species following infection (Dimopoulos et al., 1998; Luckhart et al., 1998) and may also be related to physiological functions such as increased hemolymph clearance, which support previous evidence for NOS involvement in epithelial fluid transport in *Drosophila* (Dow et al., 1994). Other lines of evidence of NOS involvement in insect immune reactions include the induction of NOS activity in a lepidopteran hemocyte cell line following bacterial infection and bacterial lipopolysaccharide presentation (Weiske and Wiesner, 1999). Among all single-cell transcriptomes consulted in this study, we note that hemocytes are constituents of datasets derived from antennal tissue, which further suggests that NO may act as a multifunctional messenger at the antennal level. Synthesis of NO in response to bacterial challenge is exhibited in olfactory mucosae of rats, where olfactory ensheathing cells produce NO as part of the innate immune response (Harris et al., 2009).

Second, NO signaling may control or participate in OSNs in a non-olfactory transduction capacity, therefore remaining undetected during odor response measurements, such as in regulating gene expression, a broad consequence of the NO signaling pathway in biological systems (Bogdan, 2001; Pfeilschifter et al., 2001; Hemish et al., 2003). As an example, circadian olfactory rhythms have been reported in insect antennae and are dependent on an autonomously oscillating regulatory gene network that establishes a daily olfactory rhythm (Tanoue et al., 2004; Schuckel et al., 2007; Flecke and Stengl, 2009; Schendzielorz et al., 2015). The involvement of NO as a key regulator establishing circadian rhythmicity is also well described in disparate models (Melo et al., 1997; Mitome et al., 2001; Tunçtan et al., 2002; Kunieda et al., 2008; Mitter et al., 2010; Plano et al., 2010; Machado-Nils et al., 2013; Gage and Nighorn, 2014), including *Drosophila* glia and neurons (Kozlov et al., 2020) and in sensory structures such as avian photoreceptors, which host circadian phase-dependent NO-mediated regulation of ion channel activity (Ko et al., 2013), and may constitute a prospective antennal role for further investigation. Curiously, clock genes such as period (per) are present in a wide variety of cell types in the antenna including OSNs and their support cells and transcript abundance corresponds to daily rhythms of pheromone response in moths (Schuckel et al., 2007; Merlin et al., 2016). Genes orthologous to per (e.g., period circadian regulator 1, Per1) have been shown to co-express with NOS in neurons, and where NO synthesis has been suggested to be controlled by circadian clock mechanisms in retinal (amacrine) neurons of rats (Zhang et al., 2005). Whether latent antennal NO signaling comes as a result or determines the apparent circadian pacemaking in insect antennae presents an interesting avenue for further research. Since we did not systematically investigate responses at different times of the circadian cycle, an olfactory response effect function may have remained obscured.

Third, NO signaling could also candidate as a messenger among non-neuronal cells of the antenna, which remain untargeted in studies focusing on imaging sensory neurons. Support cells co-activate along with their OSNs during an odor response, though no coupling mechanisms have yet been identified (Prelic et al., 2022). Here, we propose future studies test for the presence of NOS and sGC at the protein level in the third antennal segment. Being a near-range and instantaneous mediator, NO could fulfill the role of an activity-coupler in the multicellular architecture of the sensillum, especially given that coupled secondary pathways like the NO-cGMP cascade operate on time scale orders of magnitude longer than fast ionotropic transduction, and that instantaneity of response is likely unnecessary for support cells maintaining homeostasis of the sensillum lymph. Circumstantial evidence of NOS activity in antennal support cells exists and has been explicitly noted previously (Müller and Hildebrandt, 1995; Davies, 2000): NADPH-diaphorase NOS staining is strongest in the antenna of the *Apis mellifera* honeybee in support and/or epithelial cells, amid weak staining in sensory neurons and the antennal nerve (Müller and Hildebrandt, 1995). Relatedly, NOS is also found in non-neuronal support cells within the organ of Corti in the mammalian cochlea (Heinrich et al., 2004; Kopp-Scheinpflug and Forsythe, 2021) and as such may be an instance of functional parallelism in insect antennae. Here, we also find the expression of a potential NO signaling target, the cGMP-dependent protein kinase G family member foraging (for), localized to non-neuronal cells. In the present study, we found both Orco^+^ and Orco^−^ OSNs as well as Johnston organ (auditory) sensory neurons free of for transcripts (Supplementary Figure 2C); for mutants have been previously shown to exhibit differing olfactory behaviors measured via olfactory behavioral paradigms such as trap and T-maze assays, which were implicated as early hints that cGMP-dependent protein kinases play roles in olfactory behavior (Shaver et al., 1998). Likewise, the gene has been later implicated in olfactory habituation behavior and shown to be expressed in some cells of the antenna and its arista, as well as the antennal lobe, suggested to act as a player in olfactory adaptation to repeated olfactory stimulations (Eddison et al., 2012). If for is present in antennae but depleted in antennal neurons, we expect it to pose as a potential candidate target for latent NO signaling in non-neuronal cells of the antenna.

In sum, we find no evidence for NO signaling involvement in the *Drosophila* olfactory response but rather find positive antennal expression of NO-cGMP pathway machinery. Whether autocrine or paracrine NO signals are indeed present in the *Drosophila* antenna, and what biological function the pathway may ostensibly contribute to, constitute interesting unanswered avenues for future research. In contrast to findings in other insect model organisms, we also describe multiple observations of a lack of effect of cGMP on olfactory response modulation, which may further question how generalizable discoveries in olfactory system physiology may be among the highly biodiverse insect clade.

## Data availability statement

All original contributions and existing datasets presented in the study are publicly available. All data and materials used for this study are found in the online repository at this link: https://doi.org/10.17617/3.9V6MOM.

## Author contributions

DW and SP conceptualized the study. SP performed fly rearing, crossings, open antennal preparations, calcium imaging, formal analysis, visualizations, wrote the R script for the automated processing and plotting of cation imaging data, and wrote the manuscript and produced all figures. MG performed sensillum microinjection and recording experiments and analysis thereof. SP was supervised by DW and BH. DW and BH provided comments and critical input for manuscript revisions and provided experimental resources. All authors have read and agreed to the published version of the manuscript.
