# Supplementary material for: Modulation of the NO-cGMP pathway has no effect on olfactory responses in the Drosophila antenna
Source: Front Cell Neurosci. 2023 May 25;17:1180798. doi: 10.3389/fncel.2023.1180798 (PMC10248080; doi:10.3389/fncel.2023.1180798)
Supplement: Supplementary file 1 [file Data_Sheet_1.PDF]

Supplementary Figure S1

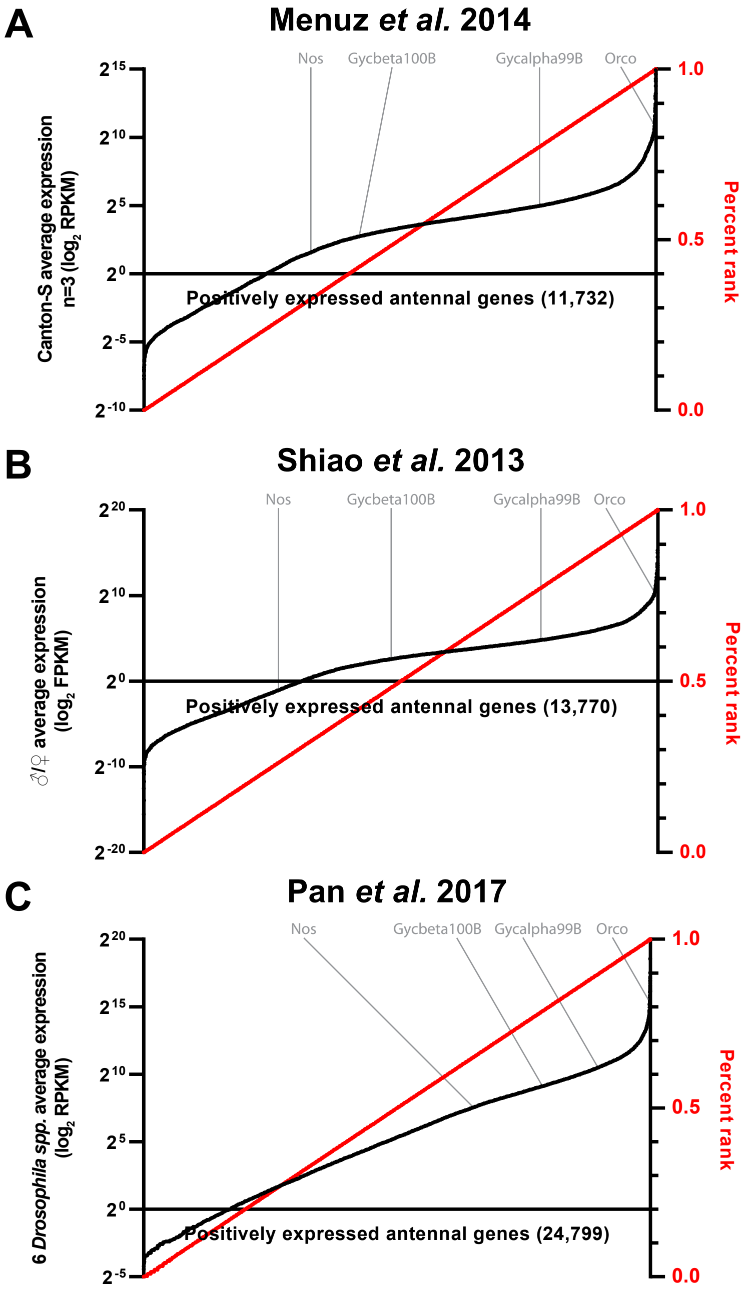

Supplementary Figure S1. Plot of abundance of all (non-zero) positively expressed genes in antennal samples across three independent RNA-seq studies, ordered from lowest to highest expressed gene (red: percentile expression among non-zero expressed genes). Genes undetected in the antenna (zero expression) are not considered. Core NO signaling pathway genes such as nitric oxide synthase (Nos) and NO-sensitive soluble guanyl cyclases (Gycα99B, Gycβ100B) are labeled alongside the highly expressed olfactory co-receptor (Orco). Core genes of interest are labeled and seem abundantly expressed in drosophilid antennae to considerable degrees relative to all other genes expressed in antennae. **(A)** Mean average expression of all detected genes across 3 antennal transcriptomes of *D. melanogaster* Canton-S antennae from Menuz *et al.* (2014) *PLoS Genetics* study. **(B)** Plot of average gene expression between male and female antennal transcriptomes of *D. melanogaster* Canton-S strain from Shiao *et al.* (2013) *Zoological Studies* study. **(C)** Mean average expression across six adult drosophilid species' antennal transcriptomes from Pan *et al.* (2017) *Scientific Reports* study; species-specific genes expressed in at least one *Drosophila* species' antennae were included, which may account for skew not observed in previous panels.

Supplementary Figure S2

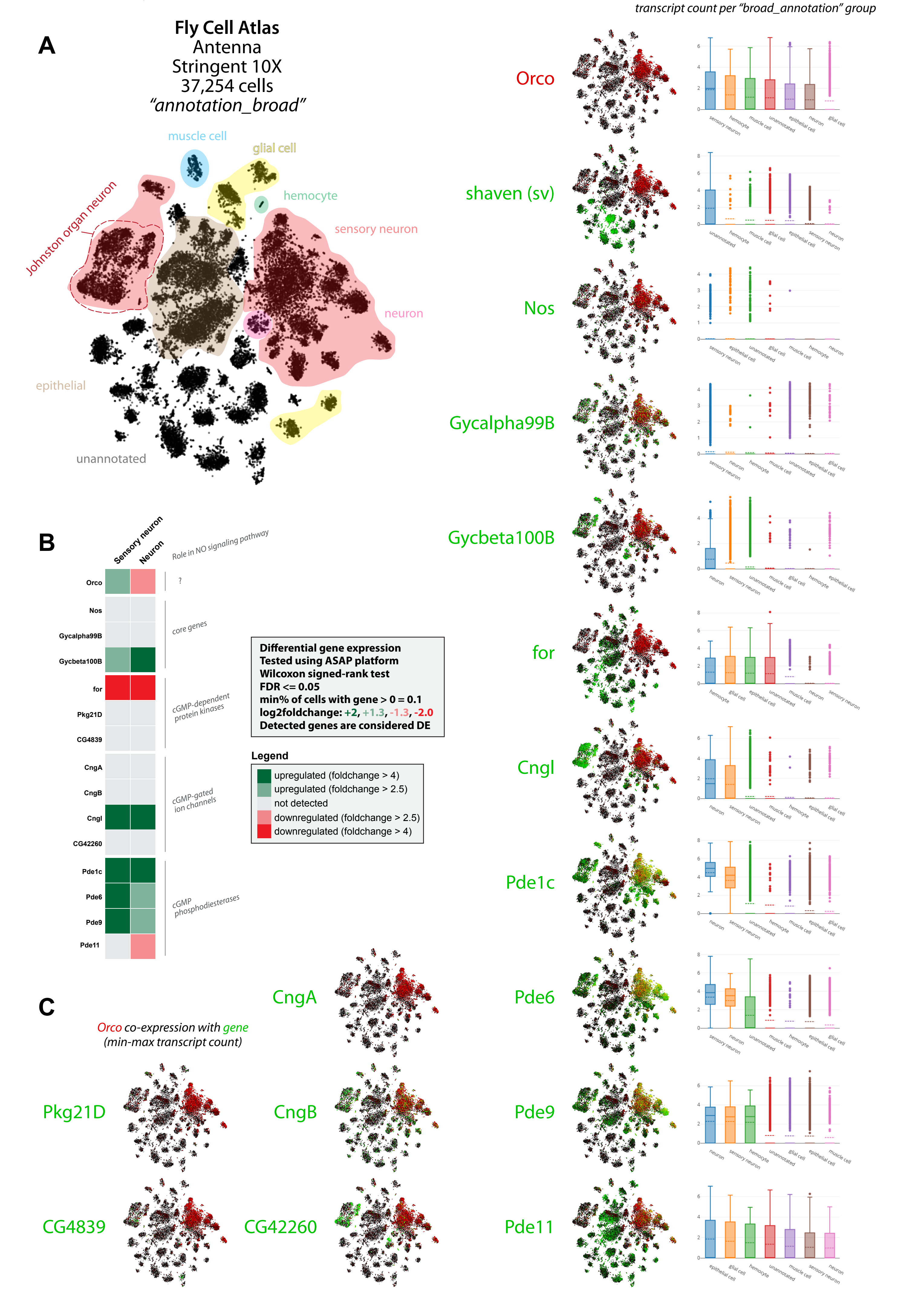

Supplementary Figure S2. Neuronal expression of genes involved as effectors in the NO signaling pathway in the *Drosophila* antenna. (A) *t*-distributed stochastic neighbor embedding (tSNE) plot of antennal cells of the Fly Cell Atlas (stringent scRNA-seq dataset derived from the 10X preparation pipeline) (Li et al., 2022). Cells cluster into broadly designated cell types, which are shaded by grouping defined under "*annotation_broad*" classification. Dashed lines outline the 'Johnston's organ neuron' cell cluster defined under the "*annotation*" classification. The tSNE visualizes the dataset which is analyzed or used to draw co-expression plots in all following panels. **(B)** Differential expression analysis employing Wilcoxon signed-rank tests (parametrization and color legend in gray boxes on right) between the 'Sensory neuron' or 'Neuron' group vs. complementary set of antennal cells, revealing specific up- or down-regulation of candidate genes involved as core participants or potential effectors of a putative NO signaling pathway. **(C)** tSNE plots colored by gene expression (min-max) for a suite of genes potentially involved NO signaling pathway, shortlisted in Table 1. A corresponding graph for select genes is displayed on the right of tSNE plots comparing transcript count across "*annotation_broad*" groups. These graphs recapitulate the differential expression results shown in panel B. The gene shaven (sv) is displayed as a non-neuronal support cell marker to illustrate cell clusters that are largely free of expression of potential genes involved in the NO signaling pathway. The cGMP-dependent protein kinase foraging (for) is largely limited in expression to support cells marked with sv and is not present any neuron clusters.

Supplementary Figure S3

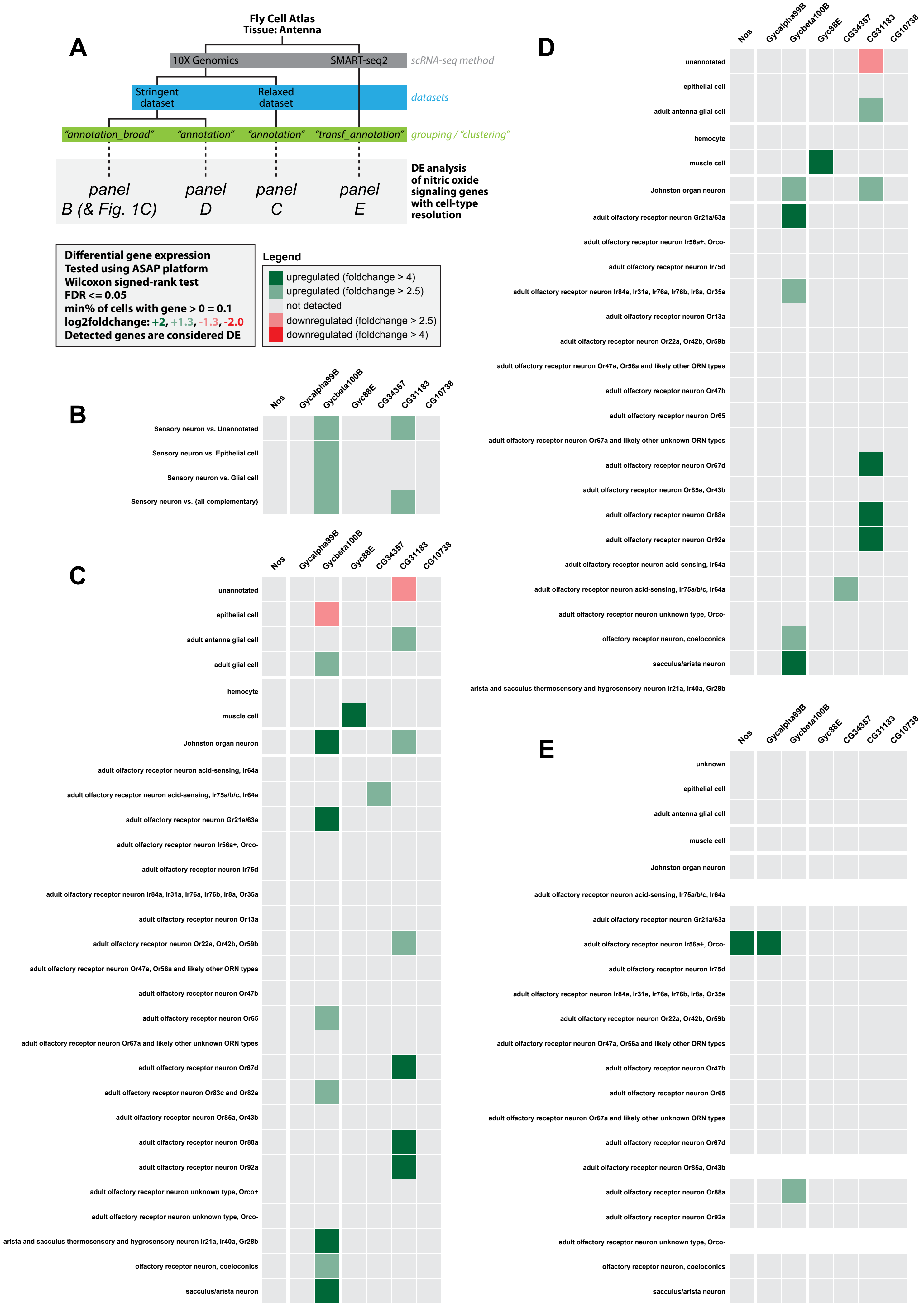

Supplementary Figure S3. Querying NO signaling pathway genes across various single-cell RNA-seq datasets. Various single-cell transcriptomes were used for differential expression (DE) analysis of some known genes involved in NO signaling, across annotated cell types. The Automated Single-cell Analysis Platform (ASAP) was used to perform Wilcoxon signed-rank tests, via Seurat, comparing two cell type classes based on expression of specific genes. **(A)** Queried antennal datasets from the Fly Cell Atlas were variously selected for comparative robustness. We looked at data originating from different single-cell isolation methods used (microfluidic droplet-based cell capture method of *10X Genomics* vs. plate-based *SMART-seq2*), within datasets generated from raw data by different data processing pipelines (*stringent* vs. *relaxed* datasets), and across different kinds of annotations of cell type, which are categorized manually by crowd annotation or through clustering (e.g. "*annotation_broad*" discriminates broadly between general cell type; *"annotation"* discriminates between cell subtype, especially within the sensory neuron class). Default parameter (excluding *fold change cutoff*) DE analysis is performed in each case. Genes detected as significantly upregulated (green) or downregulated (red) in a specific cell category compared to all other antennal cells (i.e. complementary set) are shaded based on whether they are detectable at *fold change cutoff* > 2 (dark), or > 1.3 (light). Undetected genes in DE analysis are colored gray. All other parameters were defaulted, as follows: *minimum % of cells with gene > 0* = 0.1 (10%); *false detection rate limit* = 0.05; *min%diff* = NULL; *max cells per group* = NULL. Genes considered: Nos (*Drosophila* nitric oxide synthase), Gycα99B and Gycβ100B (the nitric oxide-sensitive soluble guanyl cyclases), Gyc88E (atypical soluble guanyl cyclase), and CG34357, CG31183 and CG10738 (membrane-associated guanyl cyclase receptors, not known for being nitric oxide-sensitive). **(B)** DE analysis of genes between sensory neuron cell class vs. all other non-neuronal support cell sets, including a comparison with the sensory neuron-complementary set, i.e. all non-sensory neuron antennal cells (as annotated in *"annotation_broad"*). Dataset used: 10X Genomics-derived stringent dataset. **(C)** DE analysis of genes across all *"annotation"* cell groups vs. their respective complementary set. Dataset used: 10X Genomics-derived relaxed dataset. **(D)** Same analysis as panel C performed on the stringent sister dataset. **(E)** DE analysis on dataset generated by SMART-seq2 approach; here each cell class (defined in the SMART-seq2-specific *"trans_annotation"*) is compared against its respective complementary set, as before.

Supplementary Figure S4

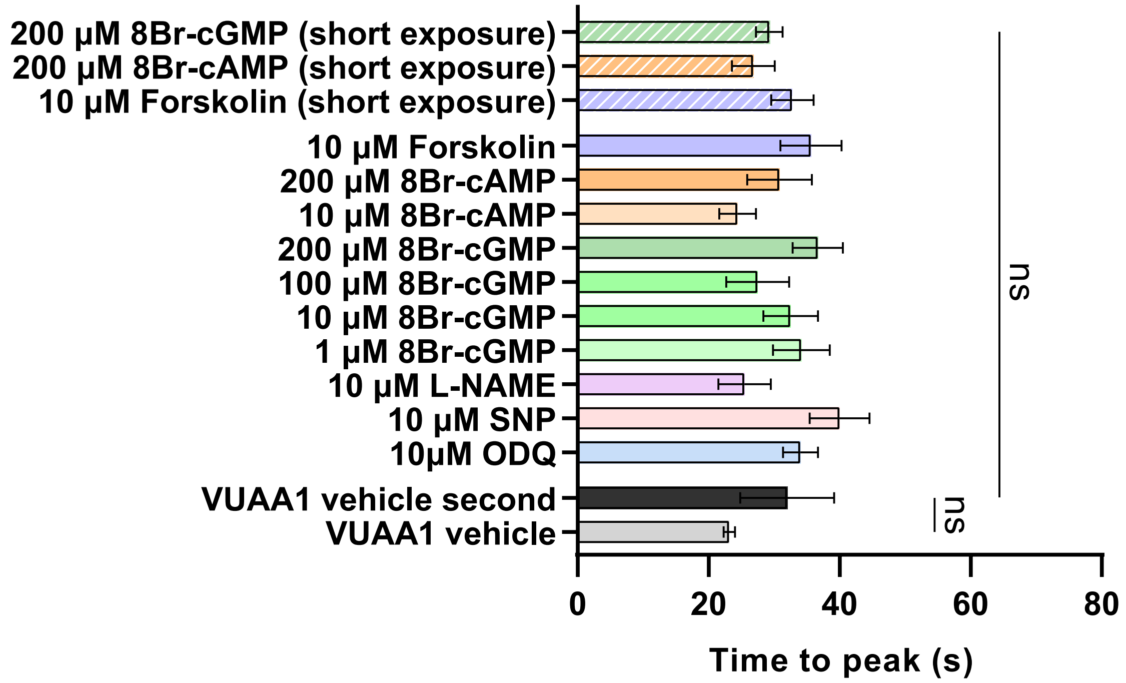

Supplementary Figure S4. Response latency kinetics across different treatments. Bars indicates mean averages ± SEM of OSN responses recorded during Ca^{2+} imaging of antennal preparations. Unpaired non-parametric ANOVA (Kruskal-Wallis test with Dunn's multiple comparison test) was used to compare time-to-peak for all responses across all independent replicates. All comparisons are statistically insignificant, indicating similarity in response between experiments and various stimulations. Analysis is based on data recorded with a 5 s temporal resolution due to use of live microscopy with a 5 s imaging cycle interval.

Supplementary Figure S5

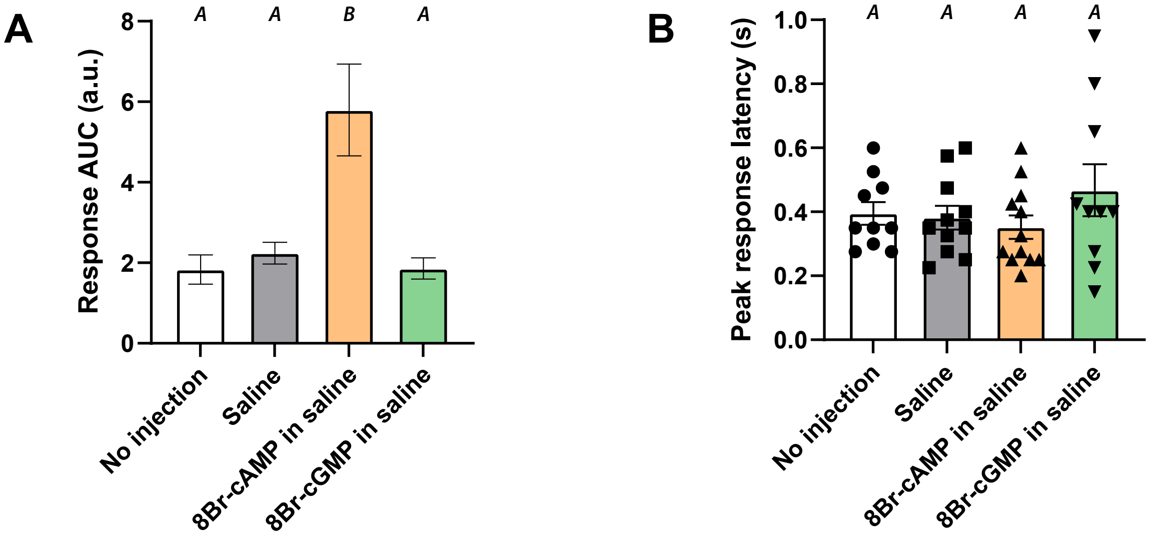

Supplementary Figure S5. Microinjection of 8Br-cAMP shows increased responses on a short temporal scale as assayed by single sensillum recording. (A) Response quantification of data shown in panel A, Figures 4E and 4F, and responses in the presence of cAMP reported previously (Getahun et al., 2013). For response frequency time plots, the area under the curve (AUC) was obtained as approximation of total number of spikes within the immediate response window (defined as 500 ms during and 1000 ms following stimulation with 10^{-5} ethyl butyrate). Errors indicate standard error of the mean. Ordinary one-way ANOVA with Dunnett's multiple comparison test was performed to compare bars. Variables labeled above with different letters are statistically significant with $p < 0.05$. **(B)** Time to reach peak response (latency) for all ethyl butyrate responses recorded via single sensillum recording, shown in panel A, Figures 4E and 4F and data reported previously (Getahun et al., 2013). For each replicate (data point), the latency from stimulus onset to maximum frequency was calculated and plotted. Errors indicate SEM. For statistical comparison, the Kruskal-Wallis test followed by Dunn's multiple comparison test was used. No treatment is significantly different from any other in peak response latency.
